# Nitro-Substituted Dipyrrolyldiketone BF_2_ Complexes as Electronic-State-Adjustable Anion-Responsive π-Electronic Systems

**DOI:** 10.3390/molecules26030595

**Published:** 2021-01-23

**Authors:** Atsuko Kuno, Hiromitsu Maeda

**Affiliations:** Department of Applied Chemistry, College of Life Sciences, Ritsumeikan University, Kusatsu 525–8577, Japan; sb0027hf@ed.ritsumei.ac.jp

**Keywords:** π-electronic systems, pyrrole derivatives, nitro groups, anion binding

## Abstract

Nitro-substituted π-electronic molecules are fascinating because of their unique electronic and optical properties and the ease of their transformation into various functional derivatives. Herein, nitro-introduced dipyrrolyldiketone BF_2_ complexes as anion-responsive π-electronic molecules were synthesized, and their electronic properties and anion-binding abilities were investigated by spectroscopic analyses and theoretical studies. The obtained nitro-substituted derivatives showed solvent-dependent UV/vis spectral changes and high anion-binding affinities due to the easily pyrrole-inverted conformations and polarized pyrrole NH sites upon the introduction of electron-withdrawing moieties.

## 1. Introduction

**Scheme 1:**
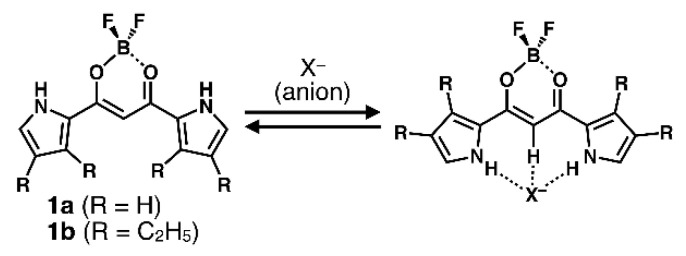
Dipyrrolyldiketone BF_2_ complexes **1a**,**b** and their anion-binding mode (X^−^: various anions such as halide, acetate, etc.).

Appropriately designed π-electronic systems have unique electronic and optical properties and assembling behavior [1,2,3,4,5,6,7]. As pyrrole-based π-electronic systems, boron-dipyrromethenes (BODIPYs) have been investigated due to high molar absorption coefficients at the visible region, high fluorescence quantum yields, and chemical and photostability. Hence, BODIPYs introducing recognition sites are used for bioimaging, sensors, and drug delivery [4,8,9,10]. Moreover, the properties of π-electronic systems can be modulated by the substituents and resulting structures. The nitro (NO_2_) group is effective for controlling the electronic states of π-electronic systems owing to its strong electron-withdrawing nature [11,12,13]. The nitro group is also a useful and important substituent in the field of synthetic chemistry. NO_2_-substituted molecules are easily converted to the corresponding amines, which are the precursors of amides and other derivatives with functional groups. It is also noteworthy that nitroarenes behave as electrophilic coupling partners for Suzuki–Miyaura coupling [14]. As the platforms for NO_2_ substitution, dipyrrolyldiketone BF_2_ complexes (e.g., **1a**,**b**, Scheme 1) have been examined as anion-responsive π-electronic molecules [15,16,17,18,19], which formed anion complexes and ion-pairing assemblies in combination with cations [20,21]. Pyrrole β-substituents of **1b** enable the facile and selective introduction of substituents at the α-positions, resulting in the synthesis of various derivatives [18,19]. The anion-binding properties depend on the substituents, such as ethyl and fluorine groups, introduced onto the pyrrole rings [16,17]. Compared to **1a**, **1b** shows less affinities for anions due to the electron-donating β-ethyl substituents. In this study, the introduction of nitro group(s) at the pyrrole α-positions was investigated for modulating the electronic states and anion-binding abilities.

## 2. Results and Discussion

### 2.1. Synthesis and Characterization

**Scheme 2:**
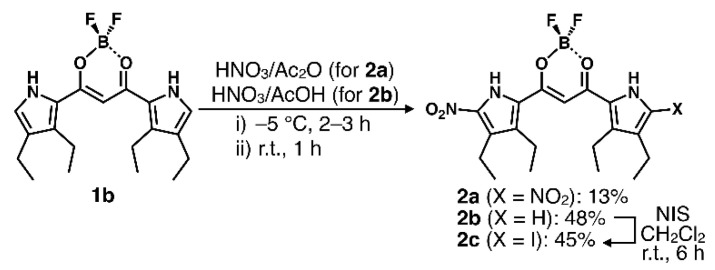
Synthesis of nitro-substituted dipyrrolyldiketone BF_2_ complexes **2a**–**c** from **1b**.

Nitration of aromatic species using nitric acid proceeds via the mechanism of electrophilic aromatic substitution (S_E_Ar) reactions. Dipyrrolyldiketone BF_2_ complex **1b** showed moderate reactivity for S_E_Ar reactions, such as iodination, at the pyrrole α-positions despite the electron-withdrawing diketone unit [18]. The modified synthesis procedure [22] was adopted to prepare dinitro-substituted **2a** as an orange solid in 13% yield from **1b** by using concentrated nitric acid and acetic anhydride. Similarly, mononitro-substituted **2b** was obtained as a red solid in 48% yield by the treatment of **1b** with concentrated nitric acid and acetic acid at −5 °C for 3 h, and subsequently, at r.t. for 1 h (Scheme 2). For the introduction of the functional group for coupling reactions [18], the reaction of **2b** with 1.1 equiv. of *N*-iodosuccinimide at r.t. for 6 h resulted in iodination at the unsubstituted pyrrole α-position to afford reddish orange **2c** in 45% yield. The ^1^H NMR spectrum of **2a** in CDCl_3_ at r.t. showed the NH signal at 10.24 ppm, whereas signals of **2b**,**c** were observed at 10.18/9.59 and 10.14/9.63 ppm, respectively. Given the pyrrole NH signal of **1b** at 9.31 ppm [17], the nitro group(s) caused the pyrrole NH signals to shift downfield. Furthermore, the signals of pyrrole β-CH_2_ in the spectra of **2a**–**c** were observed at 2.87/2.83, 2.86/2.81/2.78/2.50, and 2.86/2.83/2.78/2.46 ppm, respectively, while those of **1b** appeared at 2.78 and 2.48 ppm, suggesting the electron-withdrawing effect of the nitro groups. The bridging CH signals, 6.83, 6.64, and 6.58 ppm for **2a**–**c**, respectively, were also influenced by the nitro substitution in comparison with **1b** (6.48 ppm) [17]. 

**Figure 1:**
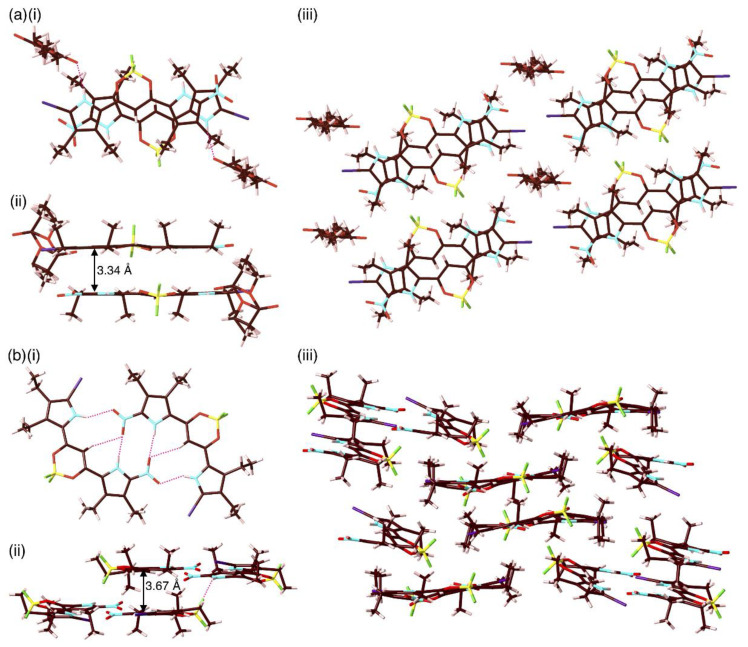
Single-crystal X-ray structures of (**a**) **2c**-tri and (**b**) **2c**-mono as (**i**) top and (**ii**) side views and (**iii**) packing diagrams. Atom color codes: brown, pink, yellow, cyan, red, green, and purple refer to carbon, hydrogen, boron, nitrogen, oxygen, fluorine, and iodine, respectively.

Solid-state structures of **2c** were revealed by single-crystal X-ray analysis (Figure 1). **2c** formed two types of crystal pseudo-polymorphs, both of which were obtained by vapor diffusion of cyclohexane into an EtOAc solution. Polymorph **2c**-tri adopted a triclinic crystal system (*P*$\bar{1}$) containing face-to-face dimers, in which non-inverted pyrrole NH formed intermolecular hydrogen bonding with a disordered EtOAc molecule with the N(−H)···O distance of 2.85 Å (Figure 1a). The **2c** molecule in **2c**-tri had a planar geometry, whose mean-plane deviation was estimated to be 0.0741 Å (19 atoms). The packing structures included π–π stacking between two pyrrole rings with a stacking distance of 3.34 Å. In contrast, polymorph **2c**-mono existed as a monoclinic crystal system (*P*2_1_/*c*) containing dimers, in which the pyrrole rings were inverted and the iodopyrrole and nitropyrrole units were in the same plane with multiple intermolecular hydrogen bonds (Figure 1b). The less stable pyrrole-inverted conformation (vide infra) in **2c**-mono was stabilized by dimerization with multiple hydrogen bonds of pyrrole NH and bridging CH with the nitro oxygen: the N(–H)···O and C(–H)···O distances were 2.89/2.90/2.97 and 3.41/3.44 Å, respectively. **2c**-mono also showed intermolecular hydrogen-bonding interactions at a pyrrolyl N–H···F(–B) with an N(–H)···F distance of 2.88 Å. The **2c** molecule in **2c**-mono also adopted the planar geometry, whose mean-plane deviations were estimated as 0.129 and 0.239 Å (19 atoms for each), and π–π stacking between the pyrrole ring and the diketone unit with a stacking distance of 3.67 Å. 

### 2.2. Photophysical Properties

**Figure 2:**
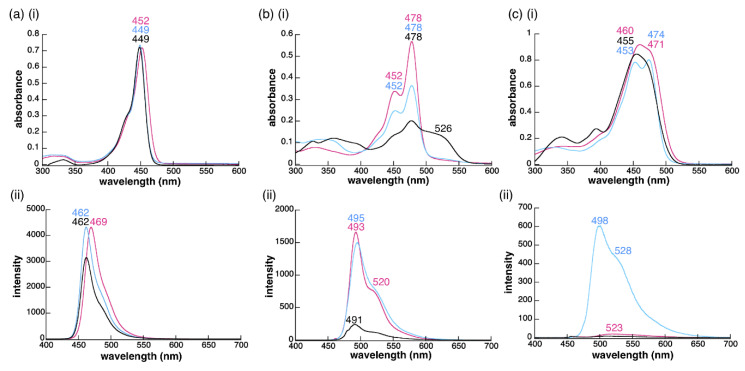
(**i**) UV/vis absorption and (**ii**) fluorescence spectra, with excitation at absorption maxima, of (**a**) **1b** (0.01 mM), (**b**) **2a** (0.01 mM), and (**c**) **2b** (0.02 mM) in toluene (cyan), CH_2_Cl_2_ (red), and THF (black).

**Figure 3:**
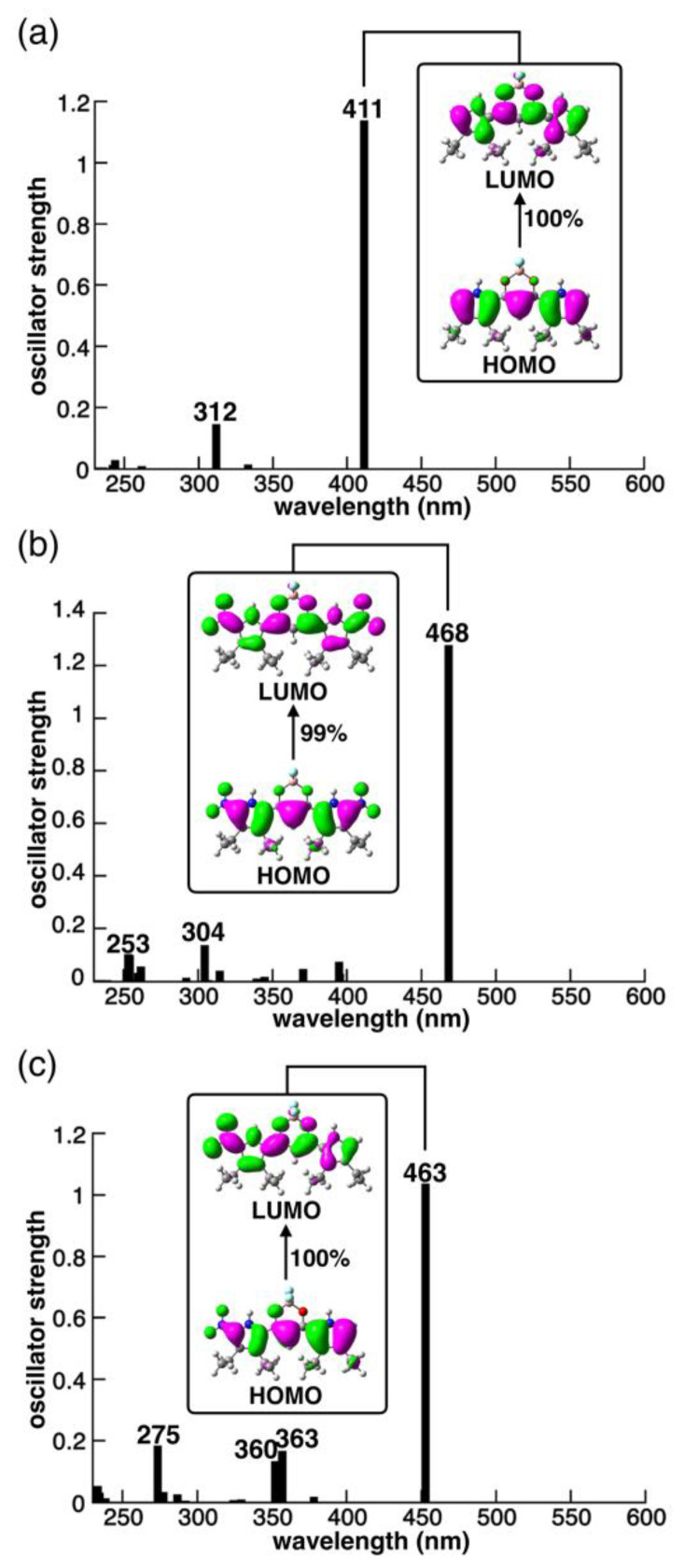
TD-DFT-based UV/vis absorption stick spectra of (**a**) **1b**, (**b**) **2a**, and (**c**) **2b** with the transitions correlated with molecular orbitals estimated at CPCM-B3LYP/6-31+G(d,p) (CH_2_Cl_2_)//B3LYP/6-31G(d,p).

The UV/vis absorption spectra of **2a**,**b** revealed characteristic substituent-dependent electronic properties. The UV/vis absorption spectra of **2a** showed the maxima at 478 nm (toluene and CH_2_Cl_2_) and a broad peak at 526 nm (THF), which were red-shifted compared to those in the case of **1b** (452 (CH_2_Cl_2_) and 449 nm (toluene and THF)) (Figure 2a,b(i)). On the other hand, the UV/vis absorption spectra of **2b** were different from those of **2a**, as observed in the maxima at 474 (toluene), 460 (CH_2_Cl_2_), and 455 (THF) nm (Figure 2b(i)). The fluorescence emission wavelengths (λ_em_) (and quantum yields, Φ_F_) of **2a**,**b**, excited at the absorption maxima, in toluene were 495 (0.462) and 498 nm (0.298), respectively, whereas those in CH_2_Cl_2_ were 493 (0.460) and 523 nm (0.020), respectively. Furthermore, the fluorescence emission of **2b** in THF, excited at 455 nm, was quenched, whereas that of **2a** was observed at 491 nm with a Φ_F_ of 0.073. The broad UV/vis absorption spectra of **2b** in CH_2_Cl_2_ and THF suggested intramolecular charge transfer (ICT). These observations of **2b** were consistent with the dipole moments of the solvents: 0.30, 1.14, and 1.74 D for toluene, CH_2_Cl_2_, and THF, respectively [23]. On the other hand, the UV/vis absorption spectrum of **2a** in THF showed broad and red-shifted bands, probably due to the interactions between the pyrrole NH and solvent molecules. This feature was also observed in the spectral change of **2a** upon the addition of CH_3_CO_2_^−^ as a tetrabutylammonium (TBA) salt in CH_2_Cl_2_ (vide infra). The Stokes shift of **2a** was 0.079 eV, which is smaller than **1b** (0.100 eV) [17], whereas the Stokes shift of **2b** was 0.325 eV due to ICT in CH_2_Cl_2_. The UV/vis absorption spectra of **1b** and **2a**,**b** were consistent with time-dependent density functional theory (TD-DFT)-based theoretical spectra at CPCM-B3LYP/6-31+G(d,p) in CH_2_Cl_2_ (Figure 3) [24]. The main theoretical absorption maxima of **1b** and **2a**,**b** were estimated to be 411, 468, and 463 nm, respectively, corresponding to HOMO (highest occupied molecular orbital)-to-LUMO (lowest unoccupied molecular orbital) transitions. Both the HOMO and LUMO of **1b** and **2a** were distributed over the core π-electronic unit owing to the symmetrical structures. In contrast, in the less symmetric **2b**, the HOMO was delocalized at the core π-electronic unit except for the carbonyl oxygen at the α-unsubstituted pyrrole side, whereas the LUMO was more distributed in the nitro-substituted pyrrole and diketone units. Hence, **2b** showed ICT, especially in more polar solvents. 

### 2.3. Anion-Binding Behavior

**Figure 4:**
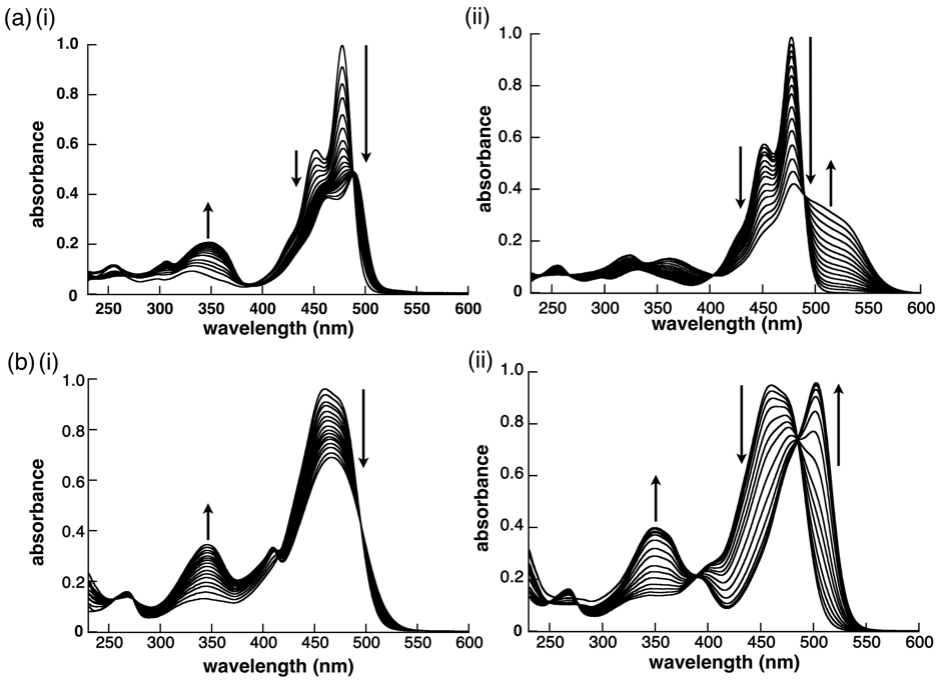
UV/vis absorption spectral changes of (**a**) **2a** (0.01 mM) and (**b**) **2b** (0.02 mM) upon the addition of (**i**) Cl^−^ and (**ii**) CH_3_CO_2_^−^ as TBA salts in CH_2_Cl_2_.

Pyrrole inversions of dipyrrolyldiketone BF_2_ complexes, whose pyrrole NH sites are oriented to the side of the carbonyl units, are required for anion binding [15,16,17,18,19]. The theoretical relative stability is correlated with the dipole magnitude and orientation of the pyrrole units. Therefore, the electronic states of pyrrole rings, according to the introduced substituents, modulate the dipole moments and resulting pyrrole-inversion behavior. Moreover, the binding constants (K_a_) are determined by the hydrogen-bonding donor strength, depending on the dipole moment of the pyrrole units. Considering these points, the anion-binding properties of **2a**,**b** were examined with UV/vis absorption spectral changes upon the addition of anions as TBA salts in CH_2_Cl_2_ solutions (0.01 mM for **2a** and 0.02 mM for **2b**). CH_2_Cl_2_ is used for good solubility for **2a**,**b** and salts and for exhibiting higher *K*_a_ values as a less polar solvent. The use of polar solvents decreases the affinities for anions due to the solvation of salts along with receptors. Upon the addition of Cl^−^, the absorption peaks in the spectrum of **2a** at 478 and 452 nm were diminished, while the peak at 350 nm became prominent due to the increasing transition dipole moment along the short axis by the conformation change from anion-free to anion-binding states (Figure 4a(i)). Similarly, in the spectrum of **2b**, the absorptions at 471 and 460 nm decreased concomitantly with an increase in the absorption of 340 nm (Figure 4a(ii)). On the other hand, the addition of CH_3_CO_2_^−^ to **2a** induced a spectral change different from those observed upon the addition of Cl^−^, where a new absorption peak around 526 nm increased while those at 478 and 452 nm decreased (Figure 4b(i)). Similar to the case of **2a**, drastic spectral changes were observed due to CH_3_CO_2_^−^ binding with **2b**, leading to new absorption peaks at around 503 and 350 nm, with decreasing peaks at 471 and 460 nm (Figure 4b(ii)). The increasing absorption peaks at 526 and 503 nm for **2a**,**b**, respectively, are derived from the changes in the electronic states. CH_3_CO_2_^−^ as a basic anion strongly interacts with pyrrole NH and induces the more polarized structures. The different basicities of Cl^−^ and CH_3_CO_2_^−^ were sensitive to the interaction strength with the more polarized nitropyrrole-NH of **2a**,**b**, resulting in different absorption spectral changes and electronic states. The K_a_ values of **2a**,**b** for Cl^−^, Br^−^, and CH_3_CO_2_^−^ (Table 1) were estimated to be 190,000, 11,000, and >10^6^ M^−1^ for **2a** and 27,000, 2800, and >10^6^ M^−1^ for **2b**, respectively, which were larger than the corresponding values for **1b** (6800, 1200, and 210,000 M^−1^, respectively) [17]. The K_a_ values were augmented with the increase in the nitropyrrole-NH units that were more effectively included for hydrogen bonding with anions. 

**Figure 5:**
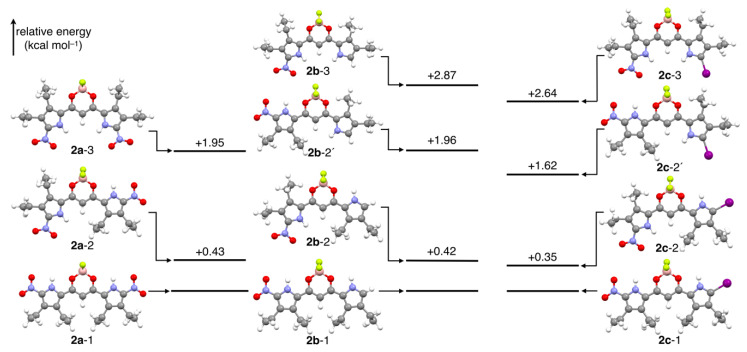
Energy diagram of diverse conformations of **2a** (**left**), **2b** (**middle**), and **2c** (**right**) calculated at the B3LYP level by using the 6-31G(d,p) basis set for C, H, B, N, O, and F and the LanL2DZ basis set for I. The relative energies (kcal/mol) are shown, and the energy standards are set to the most stable conformations of respective derivatives (**2a**-1, **2b**-1, and **2c**-1).

Theoretical studies of **2a**–**c** at the B3LYP level by using the 6-31G(d,p) basis set for C, H, B, N, O, and F and the LanL2DZ basis set for I [24] (Figure 5) suggested the stabilities of pyrrole-inverted conformations. Three conformations for **2a** and four conformations for **2b**,**c** were examined according to the orientations of pyrrole rings for the discussion of their relative energies. Singly pyrrole-inverted conformation **2a**-2 and doubly pyrrole-inverted conformation **2a**-3 were less stable than pyrrole-non-inverted conformation **2a**-1 by 0.43 and 1.95 kcal/mol, respectively. On the other hand, **2b**-2, **2b**-2′, and **2b**-3 were less stable than **2b**-1 by 0.42, 1.96, and 2.87 kcal/mol, respectively, and **2c**-2, **2c**-2′, and **2c**-3 were less stable than **2c**-1 by 0.35, 1.62, and 2.64 kcal/mol, respectively. Compared to **1b**, with lesser stabilities of 2.20 and 4.98 kcal/mol for the singly and doubly pyrrole-inverted conformations, respectively, **2a**–**c** can easily invert their pyrrole rings, especially the nitropyrrole unit(s), due to the decreased dipole moments of the pyrrole rings. 

**Figure 6:**
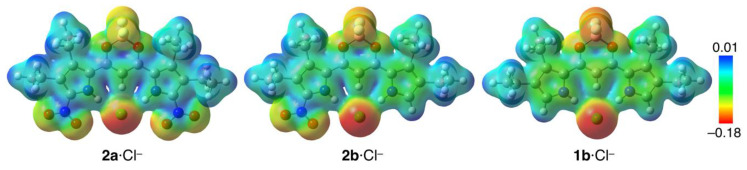
ESP mapped on the electron density isosurfaces (δ = 0.01) of **2a**,**b**·Cl^−^ and **1b**·Cl^−^ at the B3LYP-6-31+G(d,p) level.

The theoretically estimated electrostatic potential (ESP) maps of **2a**,**b**⋅Cl^−^ [24] (Figure 6) were different from that of **1b**⋅Cl^−^. The NO_2_-substituted pyrrole rings in **2a**,**b**⋅Cl^−^ are more cationic than the pyrrole rings in **1b**⋅Cl^−^, and simultaneously, more cationic BF_2_ units are observed in **2a**,**b**⋅Cl^−^ than in **1b**⋅Cl^−^ because of the strong electron-withdrawing effect of the NO_2_ units. Furthermore, Cl^−^ in **2a**⋅Cl^−^ has a smaller electron density due to the electron delocalization onto the dipyrrolyldiketone unit. These electron delocalization properties of **2a**,**b**⋅Cl^−^ are consistent with the more effective anion-binding abilities. 

**Figure 7:**
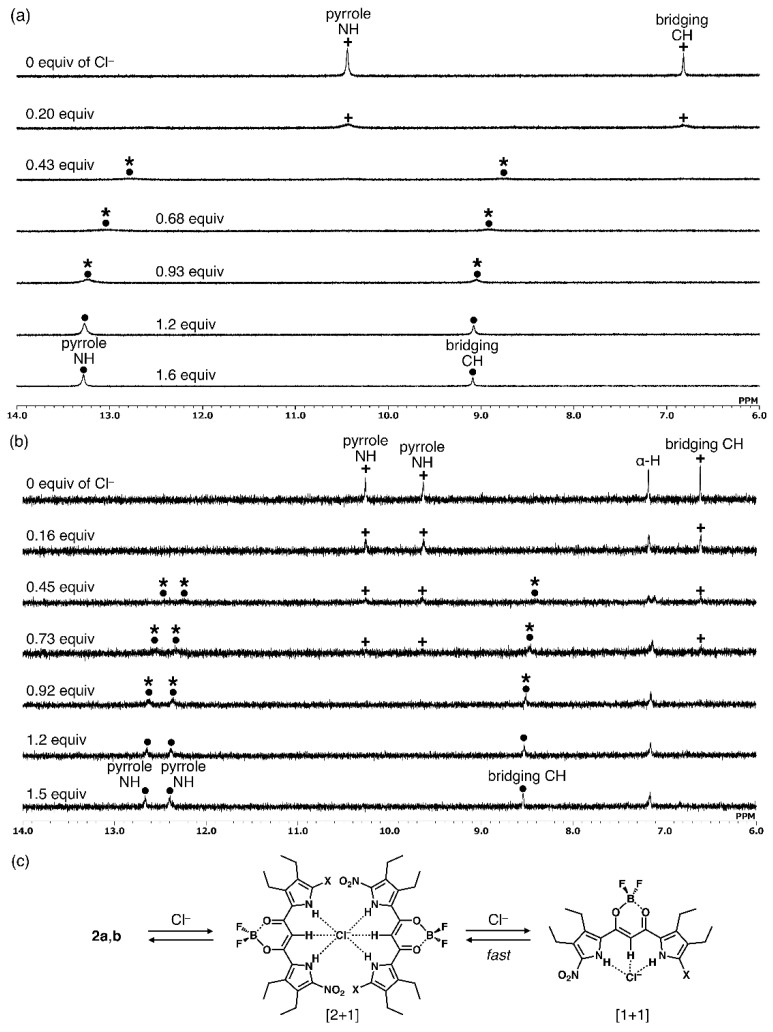
^1^H-NMR spectral changes of (**a**) **2a** and (**b**) **2b** in CD_2_Cl_2_ (1.0 mM) at −50 °C upon the addition of Cl^−^ as a TBA salt and (**c**) possible anion-binding modes of **2a**,**b** (X = NO_2_ and H, respectively). In (**a**,**b**), the signals of anion-free receptors, [1+1]-type, and [2+1]-type complexes are labeled with plus, circle, and asterisk marks, respectively.

The anion-binding modes of **2a**,**b** were investigated based on changes in their ^1^H NMR spectra upon the addition of Cl^−^ as a TBA salt in CD_2_Cl_2_ at 20 and −50 °C (Figure 7). In the case of **2a**, the addition of small amounts of Cl^−^ at −50 °C decreased the signals of pyrrole NH (10.44 ppm) and bridging CH (6.82 ppm). After the addition of 0.43 equiv. of Cl^−^, the signals of pyrrole NH and bridging NH first appeared in the downfield regions. Upon the addition of 1.2 equiv. of Cl^−^, the signals gradually shifted to 13.27 (pyrrole NH) and 9.08 ppm (bridging CH) (Figure 7a). On the other hand, in the case of **2b**, the addition of Cl^−^ (0.45 equiv.) at −50 °C decreased the signals of the pyrrole NH (10.26/9.63 ppm) and bridging CH (6.61 ppm). Concomitantly, the signals of Cl^−^-binding pyrrole NH and bridging CH appeared in the downfield regions, and the addition of 1.2 equiv. of Cl^−^ shifted the Cl^−^-binding pyrrole NH and bridging CH signals to 12.64/12.38 and 7.15 ppm, respectively (Figure 7b). These observations suggested the presence of small amounts of [2+1]-type Cl^−^ complexes. In contrast to those that independently appeared as independent signals between anion-free receptors and [1+1]-type complexes [19], the [2+1]-type complexes of **2a**,**b** were in fast equilibria with the corresponding [1+1]-type complexes, resulting in the coalescence into single signals that shifted downfield (Figure 7c). 

## 3. Materials and Methods

### 3.1. General Procedures

Starting materials were purchased from FUJIFILM Wako Pure Chemical Corp. (Osaka, Japan), Nacalai Tesque Inc. (Kyoto, Japan), Tokyo Chemical Industry Co., Ltd. (Tokyo, Japan), Sigma-Aldrich Co. (Tokyo, Japan), and were used without further purification unless otherwise stated. The spectroscopic data of **1a**,**b** were reported in the precedent reports [15,17]. NMR spectra used in the characterization of products **2a**–**c** were recorded on a JEOL ECA-600 600 MHz spectrometer (JEOL Ltd., Tokyo, Japan) and a Bruker AVANCE DRX-600 600 MHz spectrometer (Bruker, Massachusetts, USA). All NMR spectra were referenced to the solvent. UV-visible absorption spectra were recorded on a Hitachi U-3500 spectrometer (Hitachi High-Tech Science Corp., Tokyo, Japan). Fluorescence spectra and quantum yields were recorded on a Hitachi F-4500 fluorescence spectrometer (Hitachi High-Tech Science Corp., Tokyo, Japan) and a Hamamatsu Quantum Yields Measurements System for Organic LED Materials C9920-02 (Hamamatsu Photonics K.K., Hamamatsu, Japan), respectively. Matrix-assisted laser desorption ionization time-of-flight mass spectrometries (MALDI-TOF-MS) were recorded on a Shimadzu Axima-CFRplus (Shimadzu Corp., Kyoto, Japan). TLC analyses were carried out on aluminum sheets coated with silica gel 60 (Merck 5554). Column chromatography was performed on Wakogel C-300. 

#### 3.1.1. BF_2_ Complex of 1,3-bis(3,4-diethyl-5-nitropyrrol-2-yl)-1,3-propanedione, **2a**

Similar to the literature procedure [22], concentrated nitric acid (0.04 mL, d = 1.4 g/L, 0.86 mmol) was added in small portions to 0.68 mL of cooled acetic anhydride (−5 °C, brine/ice bath). The mixture was stirred at this temperature for 45 min, after which it was added dropwise to a cooled solution of BF_2_ complex of 1,3-bis(3,4-diethylpyrrol-2-yl)-1,3-propanedione **1b** [17] (77.9 mg, 0.215 mmol) in 1 mL of acetic anhydride. After the addition was completed (ca. 30 min), the mixture was stirred at −5 °C for 3 h, followed by stirring at r.t. for 1 h. The reaction mixture was diluted with ice and was quenched with NaHCO_3_ aq (pH ~ 5). The aqueous mixture was extracted with Et_2_O (5 × 15 mL) and the organic layer was neutralized by washing with NaHCO_3_ aq (pH ~ 8), and was dried with brine and Na_2_SO_4_. The solvent was removed under reduced pressure, and the residue was then chromatographed over a silica gel column (eluent: 25% EtOAc/*n*-hexane) and was recrystallized from CH_2_Cl_2_/*n*-hexane to give **2a** (12.6 mg, 27.9 µmol, 13%) as an orange solid. *R_f_* = 0.33 (25% EtOAc/*n*-hexane). ^1^H-NMR (600 MHz, CDCl_3_, 20 °C): *δ* (ppm) 10.24 (br, 2H, NH), 6.83 (s, 1H, CH), 2.87 (q, *J* = 7.8 Hz, 4H, C*H_2_*CH_3_), 2.83 (q, *J* = 7.8 Hz, 4H, C*H_2_*CH_3_), 1.32 (t, *J* = 7.8 Hz, 6H, CH_2_C*H_3_*), 1.23 (t, *J* = 7.8 Hz, 6H, CH_2_C*H_3_*). ^13^C{^1^H}-NMR (151 MHz, CDCl_3_, 20 °C): *δ* (ppm) 171.4, 137.9, 136.4, 129.4, 123.4, 95.8, 19.0, 17.7, 15.5, 14.5. UV/vis (CH_2_Cl_2_, λ_max_[nm] (ε, 10^4^ M^−1^cm^−1^)): 473 (10.0). Fluorescence (CH_2_Cl_2_ (1 × 10^−5^ M), λ_em_[nm] and Φ_F_ (λ_ex_[nm])): 494 and 0.39 (473). MALDI-TOF-MS: *m/z* (% intensity): 450.2 (60), 451.2 (100), 452.2 (47). Calcd for C_19_H_23_BF_2_N_4_O_6_ ([M − H]^−^): 451.16.

#### 3.1.2. BF_2_ Complex of 1-(3,4-diethyl-5-nitropyrrol-2-yl)-3-(3,4-diethylpyrrol-2-yl)-1,3-propanedione, **2b**

Similar to the literature procedure [22], concentrated nitric acid (0.10 mL, d = 1.4 g/L, 0.86 mmol) was added in small portions to 2.0 mL of cooled acetic acid (−5 °C, brine/ice bath). The mixture was stirred at this temperature for 45 min, after which it was added dropwise to a cooled solution of **1b** [17] (181 mg, 0.50 mmol) in 5.0 mL acetic acid. After the addition was completed (ca. 30 min), the mixture was stirred at −5 °C for 2 h, followed by stirring at r.t. for 1 h. The reaction mixture was diluted with ice and was quenched with NaHCO_3_ aq (pH ~ 5). The aqueous mixture was extracted with Et_2_O (5 × 15 mL), and the organic layer was neutralized by washing with NaHCO_3_ aq (pH ~ 8) and was dried with brine and Na_2_SO_4_. The solvent was removed under reduced pressure and the residue was then chromatographed over a silica gel column (eluent: CH_2_Cl_2_) and was recrystallized from CH_2_Cl_2_/*n*-hexane to give **2b** (97.3 mg, 23.9 µmol, 48%) as a red solid. *R_f_* = 0.19 (CH_2_Cl_2_). ^1^H-NMR (600 MHz, CDCl_3_, 20 °C): *δ* (ppm) 10.18 (br, 1H, NH), 9.59 (br, 1H, NH), 7.14 (d, *J* = 3.0 Hz, 1H, pyrrole-H), 6.64 (s, 1H, CH), 2.86 (q, *J* = 7.8 Hz, 2H, C*H_2_*CH_3_), 2.81 (q, *J* = 7.8 Hz, 2H, C*H_2_*CH_3_), 2.78 (q, *J* = 7.8 Hz, 2H, C*H_2_*CH_3_), 2.50 (q, *J* = 7.8 Hz, 2H, C*H_2_*CH_3_), 1.230 (t, *J* = 7.8 Hz, 3H, CH_2_C*H_3_*), 1.229 (t, *J* = 7.8 Hz, 3H, CH_2_C*H_3_*), 1.210 (t, *J* = 7.8 Hz, 3H, CH_2_C*H_3_*), 1.209 (t, *J* = 7.8 Hz, 3H, CH_2_C*H_3_*). ^13^C{^1^H}-NMR (151 MHz, CDCl_3_, 25 °C): *δ* (ppm) 170.1, 166.4, 139.2, 136.3, 132.7, 131.2, 129.5, 128.5, 124.6, 123.6, 93.9, 19.4, 18.7, 17.9, 17.8, 15.3, 14.8, 14.5 (the signals of ethyl units are overlapped). UV/vis (CH_2_Cl_2_, λ_max_[nm] (ε, 10^4^ M^−1^cm^−1^)): 460 (4.59). Fluorescence (CH_2_Cl_2_ (2 × 10^−5^ M), λ_em_[nm] and Φ_F_ (λ_ex_[nm])): 494 and 0.39 (473). MALDI-TOF-MS: *m/z* (% intensity): 405.3 (60), 406.3 (100), 407.3 (46). Calcd for C_19_H_24_BF_2_N_3_O_4_ ([M − H]^−^): 406.18.

#### 3.1.3. BF_2_ Complex of 3-(3,4-diethyl-5-iodopyrrol-2-yl)-1-(3,4-diethyl-5-nitropyrrol-2-yl)-1,3-propanedione, **2c**

According to the literature procedure [18], to a CH_2_Cl_2_ (50 mL) solution of **2b** (40.7 mg, 0.10 mmol) at r.t. was added *N*-iodosuccinimide (23.6 mg, 0.11 mmol). The mixture was stirred at r.t. for 6 h. The mixture was washed with water and was extracted with CH_2_Cl_2_, and it was dried over anhydrous MgSO_4_ and was evaporated to dryness. The residue was then chromatographed over a silica gel column (eluent: 10% EtOAc/*n*-hexane) and was recrystallized from CH_2_Cl_2_/*n*-hexane to give **2c** (24.2 mg, 25.0 µmol, 45%) as a reddish orange solid. *R*_f_ = 0.29 (10% EtOAc/*n*-hexane). ^1^H-NMR (600 MHz, CDCl_3_, 20 °C): *δ* (ppm) 10.14 (br, 1H, NH), 9.63 (br, 1H, NH), 6.58 (s, 1H, CH), 2.86 (q, *J* = 7.8 Hz, 2H, C*H_2_*CH_3_), 2.83 (q, *J* = 7.8 Hz, 2H, C*H_2_*CH_3_), 2.78 (q, *J* = 7.8 Hz, 2H, C*H_2_*CH_3_), 2.46 (q, *J* = 7.8 Hz, 2H, C*H_2_*CH_3_), 1.29 (t, *J* = 7.8 Hz, 6H, CH_2_C*H_3_*), 1.21 (t, *J* = 7.8 Hz, 3H, CH_2_C*H_3_*), 1.12 (t, *J* = 7.8 Hz, 3H, CH_2_C*H_3_*). ^13^C{^1^H}-NMR (151 MHz, DMSO-*d*_6_, 25 °C): *δ* (ppm) 169.4, 167.8, 139.3, 137.2, 134.3, 133.3, 130.1, 129.0, 127.0, 125.9, 94.5, 19.13, 19.05, 17.7, 17.3, 15.7, 15.3, 14.9, 14.3. MALDI-TOF-MS: *m/z* (% intensity): 531.2 (45), 532.2 (100), 533.2 (35). Calcd for C_19_H_23_BF_2_IN_3_O_4_ ([M − H]^−^): 532.07. This compound was unambiguously characterized by single-crystal X-ray analysis.

### 3.2. Method for Single-Crystal X-ray Analysis

Crystallographic data are summarized in the Supplementary Materials. A single crystal of **2c**-tri was obtained by vapor diffusion of cyclohexane into an EtOAc solution. The data crystal was an orange prism of approximate dimensions 0.130 mm × 0.090 mm × 0.010 mm. A single crystal of **2c**-mono as a polymorph was also obtained by vapor diffusion of cyclohexane into an EtOAc solution. The data crystal was an orange prism of approximate dimensions 0.130 mm × 0.090 mm × 0.010 mm. All of the data were collected at 93 K on a Rigaku XtaLAB P200 diffractometer with graphite monochromated Cu-Kα radiation (λ = 1.54184 Å), and the structure was solved by the direct method. In each structure, the non-hydrogen atoms were refined anisotropically. The structure was refined by a full-matrix least-squares method by using a SHELXL 2014 [25] (Yadokari-XG) [26,27]. CIF files (CCDC-2052538–2052539) can be obtained free of charge from the Cambridge Crystallographic Data Centre.

### 3.3. DFT Caluculations

DFT calculations were carried out using the *Gaussian 09* program [24]. Optimized structures were calculated at the B3LYP level by using the 6-31G(d,p) basis set for C, H, B, N, O, and F and the LanL2DZ basis set for I, and TD-DFT-based theoretical spectra were calculated at CPCM-B3LYP/6-31+G(d,p) (CH_2_Cl_2_)//B3LYP/6-31G(d,p). Theoretically estimated ESP maps were calculated at B3LYP/6-31+G(d,p)//B3LYP/6-31G(d,p).

## 4. Conclusions

In this study, mono- and dinitro-substituted dipyrrolyldiketone BF_2_ complexes were synthesized. One of the obtained nitro-substituted derivatives provided single crystals suitable for X-ray analysis, adopting an unfavorable pyrrole-inverted-conformation in the solid state due to the decreased dipole moment of the pyrrole rings and multiple hydrogen bonds. Theoretical studies revealed that mono-nitro-substituted derivative had localized molecular orbitals, resulting in ICT in more polar solvents. Furthermore, mono- and dinitro-substituted derivatives showed high anion-binding affinities due to the easily pyrrole-inverted conformations preorganized for anion binding and polarized pyrrole NH sites upon the introduction of electron-withdrawing moieties at the pyrrole α-positions. Further modification of the introduced nitro groups would provide a variety of anion-responsive π-electronic molecules that can act as chemical sensors and building units of ion-pairing assemblies. 

## Figures and Tables

**Table 1:** Binding constants (K_a_, M^−1^) of **2a**,**b** and **1b** [17] as a reference for various anions in CH_2_Cl_2_.

	2a	2b	1b
Cl^−^	190,000	27,000	6800
Br^−^	11,000	2800	1200
CH_3_CO_2_^−^	>10^6^	>10^6^	210,000

## Data Availability

The data presented in this study are available in the article and supplementary materials.

## Supplementary Materials

Figures S1–S3: ^1^H and ^13^C{^1^H}-NMR spectra, Figures S4 and S5: Ortep drawings of single-crystal X-ray structures, Figure S6: Optimized structures, Figure S7: Molecular orbitals (HOMO and LUMO), Figures S8–S10: Theoretical UV/vis absorption spectra, Figures S11 and S12: UV/vis absorption spectral changes and titration plots upon the addition of anions in CH_2_Cl_2_, Figure S13: ^1^H-NMR spectral changes upon the addition of TBACl in CD_2_Cl_2_.
