# Supplementary material for: Nitro-Substituted Dipyrrolyldiketone BF2 Complexes as Electronic-State-Adjustable Anion-Responsive π-Electronic Systems
Source: Molecules. 2021 Jan 23;26(3):595. doi: 10.3390/molecules26030595 (PMC7866090; doi:10.3390/molecules26030595)

checkCIF/PLATON report

Structure factors have been supplied for datablock(s) 201110maeda4_rev

THIS REPORT IS FOR GUIDANCE ONLY. IF USED AS PART OF A REVIEW PROCEDURE FOR PUBLICATION, IT SHOULD NOT REPLACE THE EXPERTISE OF AN EXPERIENCED CRYSTALLOGRAPHIC REFEREE.

No syntax errors found. CIF dictionary Interpreting this report

Datablock: 201110maeda4_rev

```
Bond precision:  C-C = 0.0108 A          Wavelength=1.54184

Cell:          a=8.6034(2)       b=9.0805(2)      c=16.4248(4)
               alpha=99.579(2)   beta=94.035(2)   gamma=108.412(2)
Temperature:   93 K
```

	Calculated	Reported
Volume	1190.09(5)	1190.09(5)
Space group	P -1	P -1
Hall group	-P 1	-P 1
Moiety formula	C19 H23 B F2 I N3 O4, 0.5(C4 H8 O2)	C19 H23 B F2 I N3 O4, 0.5(C4 H8 O2)
Sum formula	C21 H27 B F2 I N3 O5	C21 H27 B F2 I N3 O5
Mr	577.17	577.16
Dx,g cm-3	1.611	1.611
Z	2	2
Mu (mm-1)	11.038	11.038
F000	580.0	580.0
F000’	581.20	
h,k,lmax	10,10,19	10,10,19
Nref	4039	3927
Tmin,Tmax	0.349,0.895	0.551,1.000
Tmin’	0.231	

```
Correction method= # Reported T Limits: Tmin=0.551 Tmax=1.000
AbsCorr = MULTI-SCAN

Data completeness= 0.972          Theta(max)= 64.993

R(reflections)= 0.0760( 3713)     wR2(reflections)= 0.2207( 3927)

S = 1.050                  Npar= 330
```

The following ALERTS were generated. Each ALERT has the format
test-name_ALERT_alert-type_alert-level.
Click on the hyperlinks for more details of the test.

Alert level A

```
PLAT971_ALERT_2_A Check Calcd Resid. Dens.  2.05A   From O2              4.31 eA-3
```

Author Response: The large peaks around I atom are the ghost peak derived from the ripple of fourier series truncation error.

```
PLAT971_ALERT_2_A Check Calcd Resid. Dens.  1.68A   From C42             4.23 eA-3
```

Author Response: The large peaks around I atom are the ghost peak derived from the ripple of fourier series truncation error.

```
PLAT971_ALERT_2_A Check Calcd Resid. Dens.  1.03A   From I1              4.11 eA-3
```

Author Response: The large peaks around I atom are the ghost peak derived from the ripple of fourier series truncation error.

```
PLAT971_ALERT_2_A Check Calcd Resid. Dens.  1.52A   From C42             4.00 eA-3
```

Author Response: The large peaks around I atom are the ghost peak derived from the ripple of fourier series truncation error.

```
PLAT971_ALERT_2_A Check Calcd Resid. Dens.  1.16A   From I1              3.98 eA-3
```

Author Response: The large peaks around I atom are the ghost peak derived from the ripple of fourier series truncation error.

Alert level B

```
PLAT230_ALERT_2_B Hirshfeld Test Diff for    N3       --C26      .        7.2 s.u.
PLAT971_ALERT_2_B Check Calcd Resid. Dens.  1.00A   From C35             2.72 eA-3
```

Author Response: The large peaks around I atom are the ghost peak derived from the ripple of fourier series truncation error.

```
PLAT975_ALERT_2_B Check Calcd Resid. Dens.  0.45A   From O7              1.85 eA-3
```

Alert level C

```
DIFMX02_ALERT_1_C  The maximum difference density is > 0.1*ZMAX*0.75
            The relevant atom site should be identified.
THETM01_ALERT_3_C  The value of sine(theta_max)/wavelength is less than 0.590
            Calculated sin(theta_max)/wavelength =    0.5878
PLAT029_ALERT_3_C _diffrn_measured_fraction_theta_full value Low .      0.972 Why?
PLAT094_ALERT_2_C Ratio of Maximum / Minimum Residual Density ....       2.65 Report
PLAT097_ALERT_2_C Large Reported Max.  (Positive) Residual Density       4.40 eA-3
PLAT250_ALERT_2_C Large U3/U1 Ratio for Average U(i,j) Tensor ....        2.5 Note
```

```
PLAT309_ALERT_2_C Single Bonded Oxygen (C-O > 1.3 Ang) ...........         O7 Check
PLAT342_ALERT_3_C Low Bond Precision on  C-C Bonds ...............    0.01083 Ang.
PLAT480_ALERT_4_C Long H...A H-Bond Reported H1       ..F1       .       2.59 Ang.
PLAT911_ALERT_3_C Missing FCF Refl Between Thmin & STh/L=    0.588        113 Report
PLAT971_ALERT_2_C Check Calcd Resid. Dens.  0.58A   From O7              2.35 eA-3
```

Author Response: The large peaks around I atom are the ghost peak derived from the ripple of fourier series truncation error.

```
PLAT971_ALERT_2_C Check Calcd Resid. Dens.  0.98A   From I1              2.23 eA-3
```

Author Response: The large peaks around I atom are the ghost peak derived from the ripple of fourier series truncation error.

```
PLAT971_ALERT_2_C Check Calcd Resid. Dens.  0.45A   From O7              1.85 eA-3
```

Author Response: The large peaks around I atom are the ghost peak derived from the ripple of fourier series truncation error.

```
PLAT972_ALERT_2_C Check Calcd Resid. Dens.  0.97A   From I1             -1.59 eA-3
PLAT972_ALERT_2_C Check Calcd Resid. Dens.  0.92A   From I1             -1.52 eA-3
```

Alert level G

```
PLAT003_ALERT_2_G Number of Uiso or Uij Restrained non-H Atoms ...         11 Report
PLAT007_ALERT_5_G Number of Unrefined Donor-H Atoms ..............          2 Report
PLAT154_ALERT_1_G The s.u.'s on the Cell Angles are Equal ..(Note)      0.002 Degree
PLAT178_ALERT_4_G The CIF-Embedded .res File Contains SIMU Records          3 Report
PLAT186_ALERT_4_G The CIF-Embedded .res File Contains ISOR Records          4 Report
PLAT300_ALERT_4_G Atom Site Occupancy of O6         Constrained at        0.5 Check
PLAT300_ALERT_4_G Atom Site Occupancy of O7         Constrained at        0.5 Check
PLAT300_ALERT_4_G Atom Site Occupancy of C50        Constrained at        0.5 Check
PLAT300_ALERT_4_G Atom Site Occupancy of C51        Constrained at        0.5 Check
PLAT300_ALERT_4_G Atom Site Occupancy of C52        Constrained at        0.5 Check
PLAT300_ALERT_4_G Atom Site Occupancy of C53        Constrained at        0.5 Check
PLAT300_ALERT_4_G Atom Site Occupancy of H24        Constrained at        0.5 Check
PLAT300_ALERT_4_G Atom Site Occupancy of H25        Constrained at        0.5 Check
PLAT300_ALERT_4_G Atom Site Occupancy of H26        Constrained at        0.5 Check
PLAT300_ALERT_4_G Atom Site Occupancy of H27        Constrained at        0.5 Check
PLAT300_ALERT_4_G Atom Site Occupancy of H28        Constrained at        0.5 Check
PLAT300_ALERT_4_G Atom Site Occupancy of H29        Constrained at        0.5 Check
PLAT300_ALERT_4_G Atom Site Occupancy of H30        Constrained at        0.5 Check
PLAT300_ALERT_4_G Atom Site Occupancy of H31        Constrained at        0.5 Check
PLAT302_ALERT_4_G Anion/Solvent/Minor-Residue Disorder (Resd  2  )       100% Note
PLAT380_ALERT_4_G Incorrectly? Oriented X(sp2)-Methyl Moiety .....        C53 Check
PLAT431_ALERT_2_G Short Inter HL..A Contact  I1       ..O2       .       3.18 Ang.
                                                    x,y,1+z  =      1_556 Check
PLAT789_ALERT_4_G Atoms with Negative _atom_site_disorder_group  #         14 Check
PLAT860_ALERT_3_G Number of Least-Squares Restraints .............        114 Note
PLAT883_ALERT_1_G No Info/Value for _atom_sites_solution_primary .     Please Do !
PLAT909_ALERT_3_G Percentage of I>2sig(I) Data at Theta(Max) Still        89% Note
PLAT933_ALERT_2_G Number of OMIT Records in Embedded .res File ...          4 Note
PLAT941_ALERT_3_G Average HKL Measurement Multiplicity ...........        3.7 Low
PLAT965_ALERT_2_G The SHELXL WEIGHT Optimisation has not Converged     Please Check
PLAT978_ALERT_2_G Number C-C Bonds with Positive Residual Density.          1 Info
```

```
  5 ALERT level A = Most likely a serious problem - resolve or explain
  3 ALERT level B = A potentially serious problem, consider carefully
 15 ALERT level C = Check. Ensure it is not caused by an omission or oversight
 30 ALERT level G = General information/check it is not something unexpected

  3 ALERT type 1 CIF construction/syntax error, inconsistent or missing data
 22 ALERT type 2 Indicator that the structure model may be wrong or deficient
  7 ALERT type 3 Indicator that the structure quality may be low
 20 ALERT type 4 Improvement, methodology, query or suggestion
  1 ALERT type 5 Informative message, check
```

It is advisable to attempt to resolve as many as possible of the alerts in all categories. Often the minor alerts point to easily fixed oversights, errors and omissions in your CIF or refinement strategy, so attention to these fine details can be worthwhile. In order to resolve some of the more serious problems it may be necessary to carry out additional measurements or structure refinements. However, the purpose of your study may justify the reported deviations and the more serious of these should normally be commented upon in the discussion or experimental section of a paper or in the "special_details" fields of the CIF. checkCIF was carefully designed to identify outliers and unusual parameters, but every test has its limitations and alerts that are not important in a particular case may appear. Conversely, the absence of alerts does not guarantee there are no aspects of the results needing attention. It is up to the individual to critically assess their own results and, if necessary, seek expert advice.

Publication of your CIF in IUCr journals

A basic structural check has been run on your CIF. These basic checks will be run on all CIFs submitted for publication in IUCr journals (*Acta Crystallographica*, *Journal of Applied Crystallography*, *Journal of Synchrotron Radiation*); however, if you intend to submit to *Acta Crystallographica Section C* or *E* or *IUCrData*, you should make sure that full publication checks are run on the final version of your CIF prior to submission.

Publication of your CIF in other journals

Please refer to the *Notes for Authors* of the relevant journal for any special instructions relating to CIF submission.

Validation response form

Please find below a validation response form (VRF) that can be filled in and pasted into your CIF.

```
# start Validation Reply Form
_vrf_DIFMX02_201110maeda4_rev
;
PROBLEM: The maximum difference density is > 0.1*ZMAX*0.75
RESPONSE: ...
;
_vrf_THETM01_201110maeda4_rev
;
PROBLEM: The value of sine(theta_max)/wavelength is less than 0.590
RESPONSE: ...
;
_vrf_PLAT230_201110maeda4_rev
;
PROBLEM: Hirshfeld Test Diff for    N3       --C26      .        7.2 s.u.
```

```
RESPONSE: ...
;
_vrf_PLAT975_201110maeda4_rev
;
PROBLEM: Check Calcd Resid. Dens.  0.45A   From O7              1.85 eA-3
RESPONSE: ...
;
_vrf_PLAT029_201110maeda4_rev
;
PROBLEM: _diffrn_measured_fraction_theta_full value Low .      0.972 Why?
RESPONSE: ...
;
_vrf_PLAT094_201110maeda4_rev
;
PROBLEM: Ratio of Maximum / Minimum Residual Density ....       2.65 Report
RESPONSE: ...
;
_vrf_PLAT097_201110maeda4_rev
;
PROBLEM: Large Reported Max.  (Positive) Residual Density       4.40 eA-3
RESPONSE: ...
;
_vrf_PLAT250_201110maeda4_rev
;
PROBLEM: Large U3/U1 Ratio for Average U(i,j) Tensor ....        2.5 Note
RESPONSE: ...
;
_vrf_PLAT309_201110maeda4_rev
;
PROBLEM: Single Bonded Oxygen (C-O > 1.3 Ang) ..........          O7 Check
RESPONSE: ...
;
_vrf_PLAT342_201110maeda4_rev
;
PROBLEM: Low Bond Precision on  C-C Bonds ..............    0.01083 Ang.
RESPONSE: ...
;
_vrf_PLAT480_201110maeda4_rev
;
PROBLEM: Long H...A H-Bond Reported H1       ..F1       .       2.59 Ang.
RESPONSE: ...
;
_vrf_PLAT911_201110maeda4_rev
;
PROBLEM: Missing FCF Refl Between Thmin & STh/L=    0.588        113 Report
RESPONSE: ...
;
_vrf_PLAT972_201110maeda4_rev
;
PROBLEM: Check Calcd Resid. Dens.  0.97A   From I1             -1.59 eA-3
RESPONSE: ...
;
# end Validation Reply Form
```

PLATON version of 18/09/2020; check.def file version of 20/08/2020

Datablock 201110maeda4_rev - ellipsoid plot

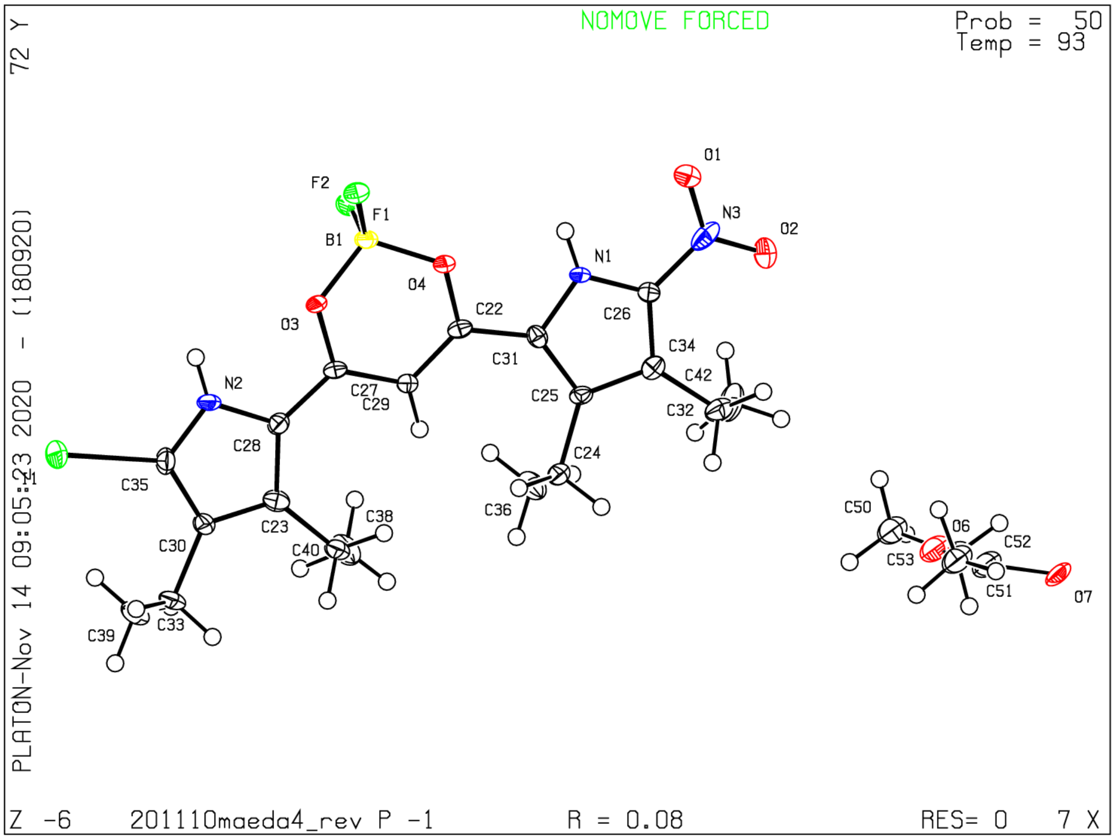

checkCIF/PLATON report

Structure factors have been supplied for datablock(s) 201110maeda

THIS REPORT IS FOR GUIDANCE ONLY. IF USED AS PART OF A REVIEW PROCEDURE FOR PUBLICATION, IT SHOULD NOT REPLACE THE EXPERTISE OF AN EXPERIENCED CRYSTALLOGRAPHIC REFEREE.

No syntax errors found.   CIF dictionary   Interpreting this report

Datablock: 201110maeda

```
Bond precision:  C-C = 0.0046 A         Wavelength=1.54184

Cell:            a=11.2903(1)     b=22.2353(2)       c=16.8402(1)
                 alpha=90         beta=98.740(1)     gamma=90
Temperature:     93 K

                Calculated                 Reported
Volume          4178.53(6)                 4178.53(6)
Space group     P 21/c                     P 21/c
Hall group      -P 2ybc                    -P 2ybc
Moiety formula  C19 H23 B F2 I N3 O4       C19 H23 B F2 I N3 O4
Sum formula     C19 H23 B F2 I N3 O4       C19 H23 B F2 I N3 O4
Mr              533.11                     533.11
Dx,g cm-3       1.695                      1.695
Z               8                          8
Mu (mm-1)       12.482                     12.482
F000            2128.0                     2128.0
F000'           2132.21
h,k,lmax        13,27,20                   13,27,20
Nref            8312                       8143
Tmin,Tmax       0.299,0.883                0.576,1.000
Tmin'           0.172

Correction method= # Reported T Limits: Tmin=0.576 Tmax=1.000
AbsCorr = MULTI-SCAN

Data completeness= 0.980          Theta(max)= 72.751

R(reflections)= 0.0310( 7739)     wR2(reflections)= 0.0849( 8143)

S = 1.034                   Npar= 549
```

The following ALERTS were generated. Each ALERT has the format
test-name_ALERT_alert-type_alert-level.
Click on the hyperlinks for more details of the test.

Alert level C

```
PLAT431_ALERT_2_C Short Inter HL..A Contact  F1       ..O5       .       2.81 Ang.
                                             x,3/2-y,-1/2+z  =      4_575 Check
PLAT431_ALERT_2_C Short Inter HL..A Contact  F1       ..N3       .       2.88 Ang.
                                             x,3/2-y,-1/2+z  =      4_575 Check
PLAT911_ALERT_3_C Missing FCF Refl Between Thmin & STh/L=    0.600         34 Report
```

Alert level G

```
PLAT007_ALERT_5_G Number of Unrefined Donor-H Atoms ..............          4 Report
PLAT083_ALERT_2_G SHELXL Second Parameter in WGHT  Unusually Large       8.65 Why ?
PLAT142_ALERT_4_G s.u. on b - Axis Small or Missing .............     0.00020 Ang.
PLAT143_ALERT_4_G s.u. on c - Axis Small or Missing .............     0.00010 Ang.
PLAT434_ALERT_2_G Short Inter HL..HL Contact I1       ..F2               3.01 Ang.
                                           2-x,-1/2+y,1/2-z  =      2_745 Check
PLAT434_ALERT_2_G Short Inter HL..HL Contact I2       ..F4               2.93 Ang.
                                           1-x,-1/2+y,1/2-z  =      2_645 Check
PLAT883_ALERT_1_G No Info/Value for _atom_sites_solution_primary .     Please Do !
PLAT912_ALERT_4_G Missing # of FCF Reflections Above STh/L=  0.600        135 Note
PLAT941_ALERT_3_G Average HKL Measurement Multiplicity ...........        3.5 Low
PLAT965_ALERT_2_G The SHELXL WEIGHT Optimisation has not Converged     Please Check
PLAT978_ALERT_2_G Number C-C Bonds with Positive Residual Density.          5 Info
```

0 **ALERT level A** = Most likely a serious problem - resolve or explain
0 **ALERT level B** = A potentially serious problem, consider carefully
3 **ALERT level C** = Check. Ensure it is not caused by an omission or oversight
11 **ALERT level G** = General information/check it is not something unexpected

1 ALERT type 1 CIF construction/syntax error, inconsistent or missing data
7 ALERT type 2 Indicator that the structure model may be wrong or deficient
2 ALERT type 3 Indicator that the structure quality may be low
3 ALERT type 4 Improvement, methodology, query or suggestion
1 ALERT type 5 Informative message, check

It is advisable to attempt to resolve as many as possible of the alerts in all categories. Often the minor alerts point to easily fixed oversights, errors and omissions in your CIF or refinement strategy, so attention to these fine details can be worthwhile. In order to resolve some of the more serious problems it may be necessary to carry out additional measurements or structure refinements. However, the purpose of your study may justify the reported deviations and the more serious of these should normally be commented upon in the discussion or experimental section of a paper or in the "special_details" fields of the CIF. checkCIF was carefully designed to identify outliers and unusual parameters, but every test has its limitations and alerts that are not important in a particular case may appear. Conversely, the absence of alerts does not guarantee there are no aspects of the results needing attention. It is up to the individual to critically assess their own results and, if necessary, seek expert advice.

Publication of your CIF in IUCr journals

A basic structural check has been run on your CIF. These basic checks will be run on all CIFs submitted for publication in IUCr journals (*Acta Crystallographica*, *Journal of Applied Crystallography*, *Journal of Synchrotron Radiation*); however, if you intend to submit to *Acta Crystallographica Section C* or *E* or *IUCrData*, you should make sure that full publication checks are run on the final version of your CIF prior to submission.

Publication of your CIF in other journals

Please refer to the *Notes for Authors* of the relevant journal for any special instructions relating to CIF submission.

PLATON version of 18/09/2020; check.def file version of 20/08/2020

Datablock 201110maeda - ellipsoid plot

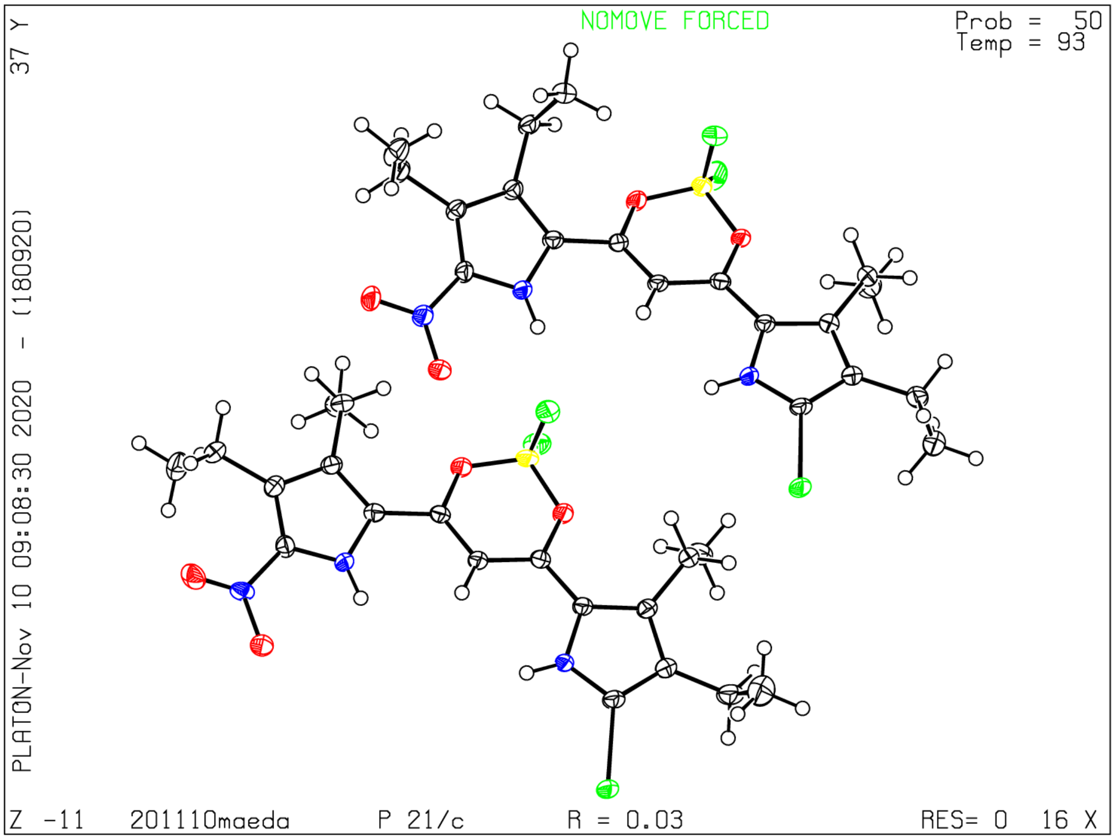

Supplement: Supplementary file 1 [file molecules-26-00595-s001.zip › NO2_kuno_HMaeda_SI/NO2_kuno_HMaeda_checkcif.pdf]
