# Supplementary material for: Nitro-Substituted Dipyrrolyldiketone BF2 Complexes as Electronic-State-Adjustable Anion-Responsive π-Electronic Systems
Source: Molecules. 2021 Jan 23;26(3):595. doi: 10.3390/molecules26030595 (PMC7866090; doi:10.3390/molecules26030595)
Supplement: Supplementary file 1 [file molecules-26-00595-s001.zip › NO2_kuno_HMaeda_SI/NO2_kuno_HMaeda_SI.pdf]

Supplementary Materials

Nitro-Substituted Dipyrrolyldiketone BF_2 Complexes as Electronic-State-Adjustable Anion-Responsive π-Electronic Systems

Atsuko Kuno and Hiromitsu Maeda*

Department of Applied Chemistry, College of Life Sciences, Ritsumeikan University, Kusatsu 525–8577, Japan, Fax: +81 77 561 2659; Tel: +81 77 561 5969; E-mail: maedahir@ph.ritsumei.ac.jp

Table of Contents

1. Spectroscopic data S2
Figure S1–3 ^{1}H NMR and ^{13}C{^{1}H} NMR spectra. S2
2. X-ray crystallographic data S5
Figure S4,5 Ortep drawings of single-crystal X-ray structures. S5
3. Theoretical study S7
Figure S6 Optimized structures. S7
Figure S7 Molecular orbitals (HOMO and LUMO). S8
Figure S8–10 Theoretical UV/vis absorption spectra. S9
Cartesian coordination of optimized structures S10
4. Anion-binding behaviors S17
Figure S11,12 UV/vis absorption spectral changes and titration plots upon the addition of anions in CH_2Cl_2. S17
Figure S13 ^{1}H NMR spectral changes upon the addition of TBACl in CD_2Cl_2. S19

1. Spectroscopic data

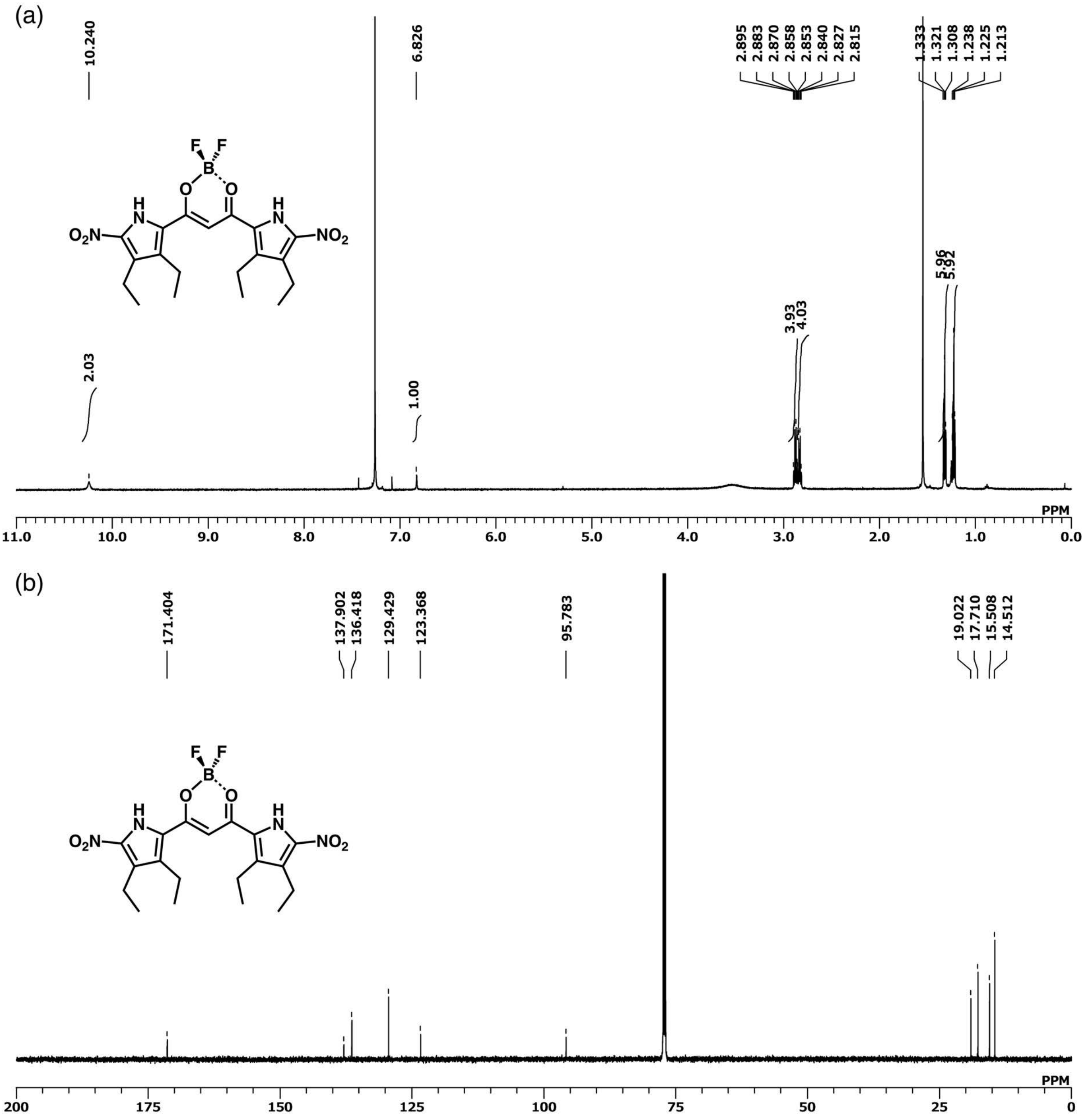

Figure S1 (a) ^{1}H NMR and (b) ^{13}C{^{1}H} NMR spectra of **2a** in $CDCl_3$ at 20 °C.

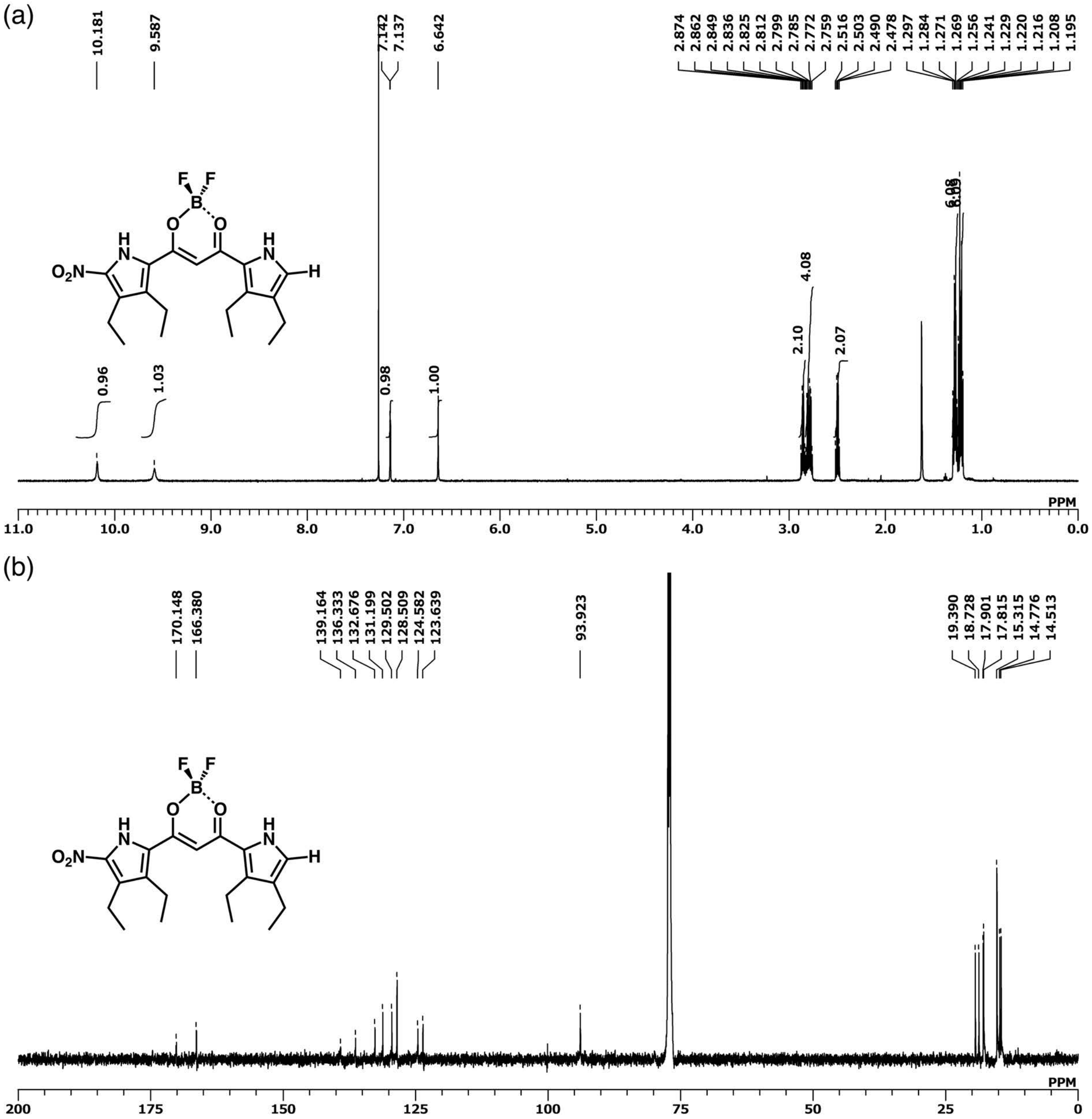

Figure S2 (a) ^{1}H NMR and (b) $^{13}C\{^{1}H\}$ NMR spectra of **2b** in $CDCl_3$ at 20 and 25 °C, respectively.

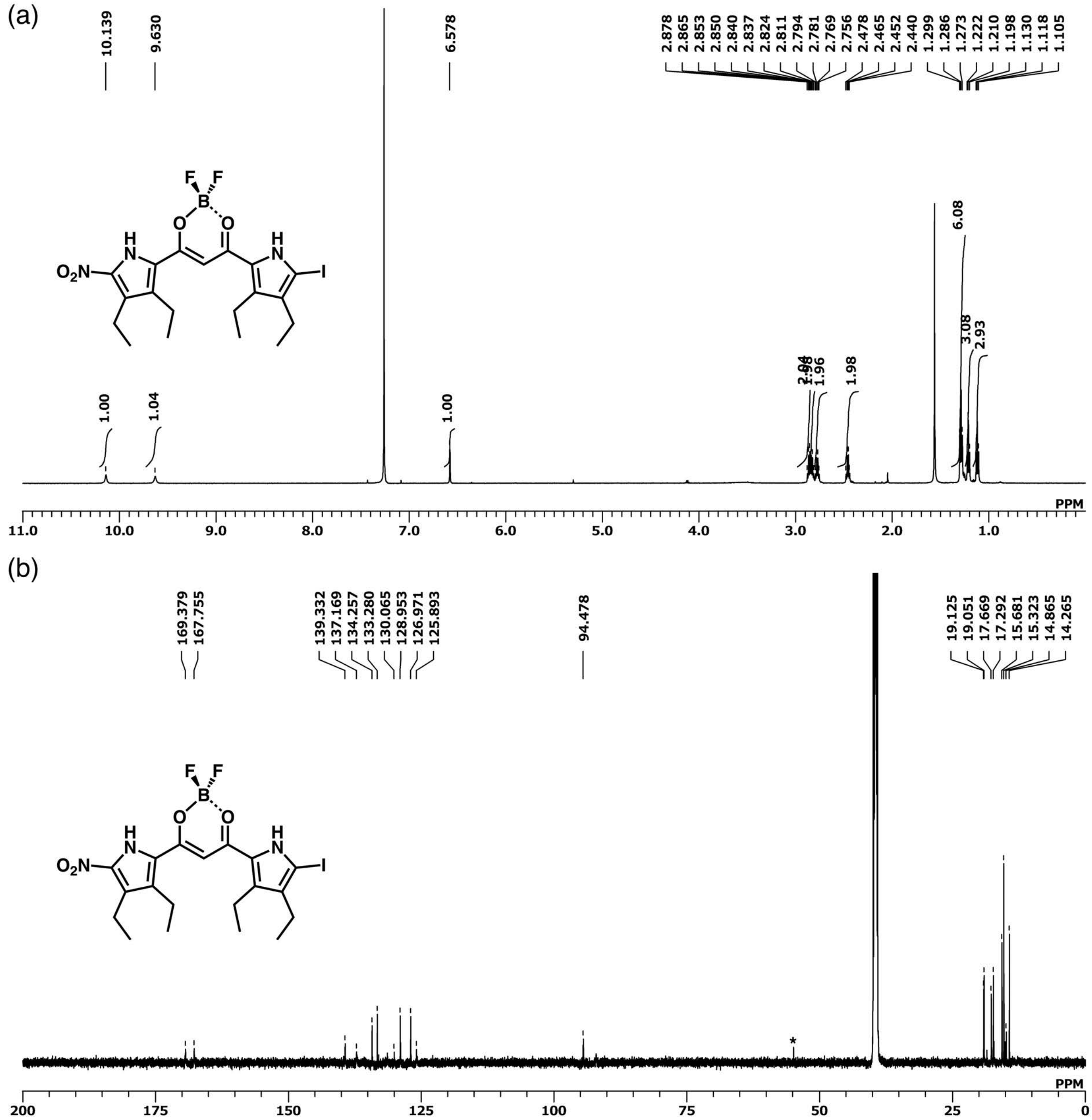

Figure S3 (a) ^{1}H NMR and (b) $^{13}C\{^{1}H\}$ NMR spectra of **2c** in $CDCl_3$ at 20 °C and DMSO-d_6 at 25 °C, respectively. The signal labeled with an asterisk mark is that of the residual CH_2Cl_2.

2. X-ray crystallographic data

Table S1 Crystallographic details.

	**2c**-tri	**2c**-mono
formula	$C_{19}H_{23}BF_2IN_3O_4 \cdot 0.5C_4H_8O_2$	$C_{19}H_{23}BF_2IN_3O_4$
fw	577.16	533.11
crystal size, mm	0.120 × 0.090 × 0.010	0.130 × 0.090 × 0.010
crystal system	triclinic	monoclinic
space group	*P*-1 (no. 2)	$P2_1/c$ (no. 14)
a, Å	8.6034(2)	11.29030(10)
b, Å	9.0805(2)	22.2353(2)
c, Å	16.4248(4)	16.84020(10)
α, °	99.579(2)	90
β, °	94.035(2)	98.7400(10)
γ, °	108.412(2)	90
V, Å^3	1190.09(5)	4178.53(6)
ρ_{calcd}, gcm^{-3}	1.611	1.695
Z	2	8
T, K	93(2)	93(2)
μ, mm^{-1} (Cu-Kα)	11.038	12.482
no. of reflns	14450	28887
no. of unique reflns	3927	8143
variables	330	549
λ, Å (Cu-Kα)	1.54184	1.54184
R_1 ($I > 2\sigma(I)$)	0.0760	0.0310
wR_2 ($I > 2\sigma(I)$)	0.2190	0.0839
GOF	1.050	1.034

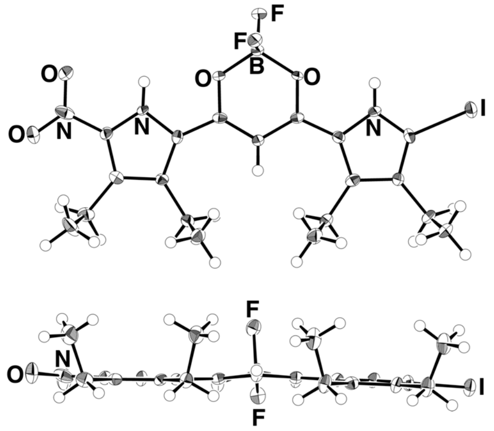

Figure S4 Ortep drawing of single-crystal X-ray structure (top and side views) of **2c**-tri. Thermal ellipsoids are scaled to the 50% probability level. Solvent molecules are omitted for clarity.

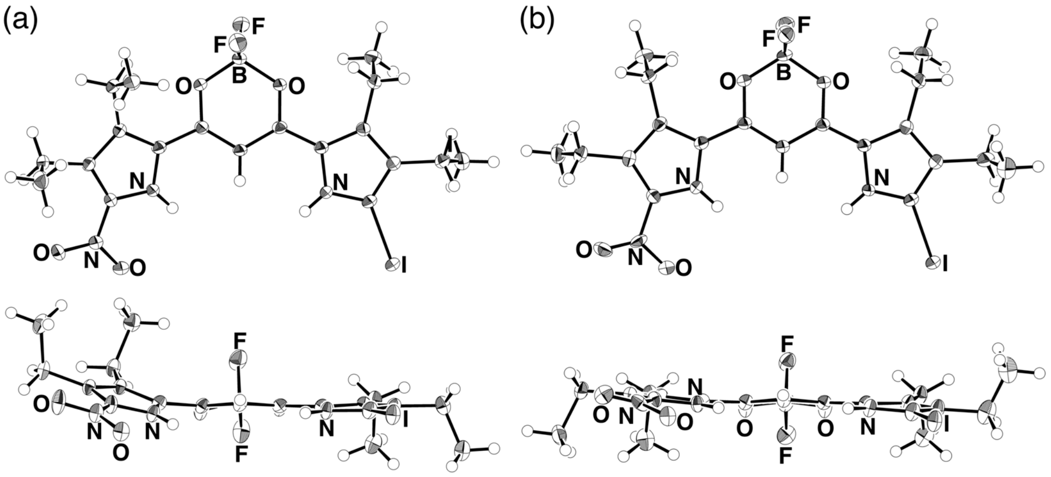

Figure S5 Ortep drawing of single-crystal X-ray structure (top and side views) of **2c**-mono ((a,b) two independent structures). Thermal ellipsoids are scaled to the 50% probability level.

3. Theoretical study

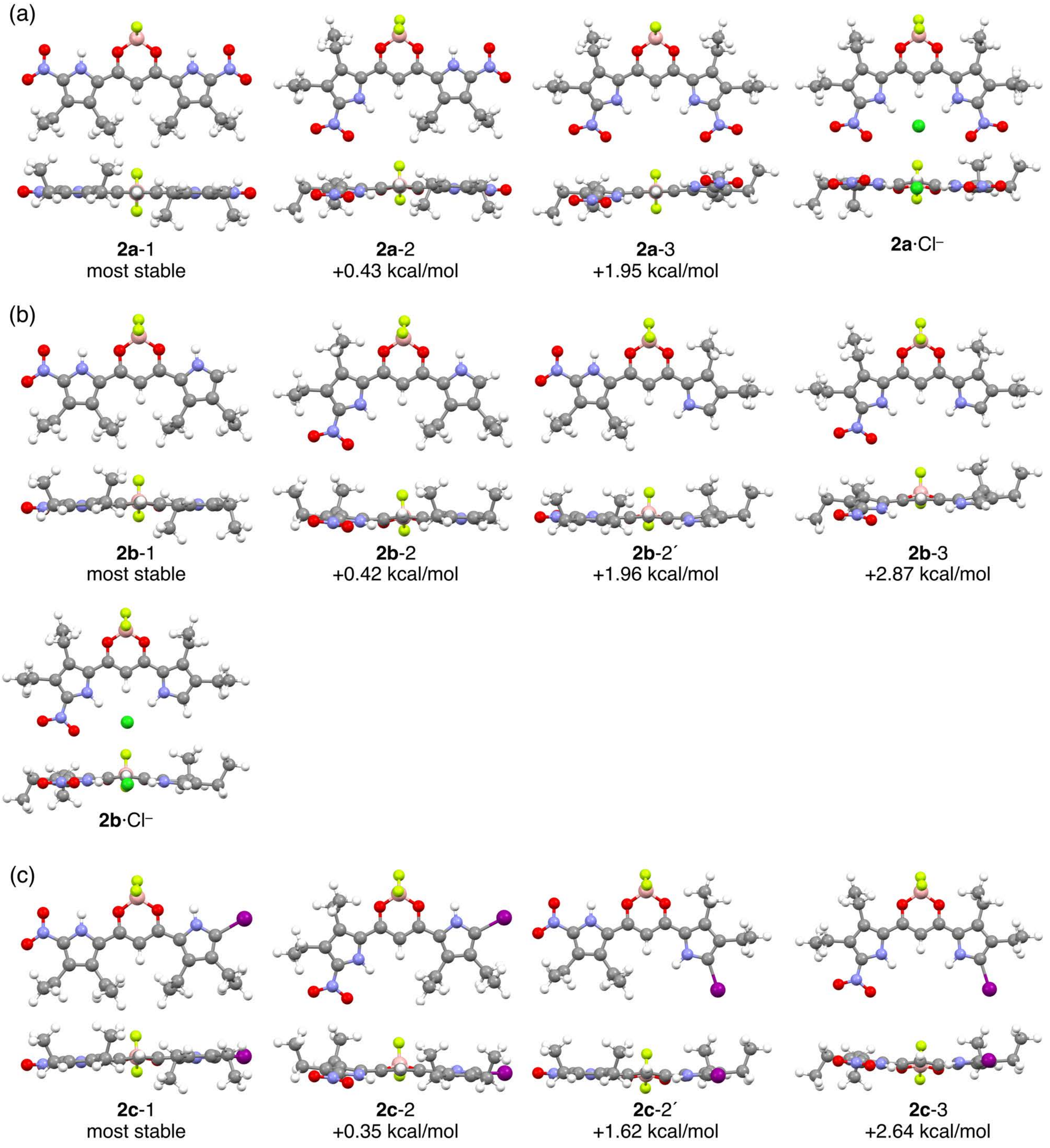

Figure S6 Optimized structures (top and side views) and relative energies of (a) **2a** (three conformations) and **2a**·Cl^-, (b) **2b** (four conformations) and **2b**·Cl^-, and (c) **2c** (four conformations) at the B3LYP level by using the 6-31G(d,p) basis set for C, H, B, N, O, and F and the LanL2DZ basis set for I (**2a**–**c**) and B3LYP/6-31+G(d,p) (Cl^- complexes).

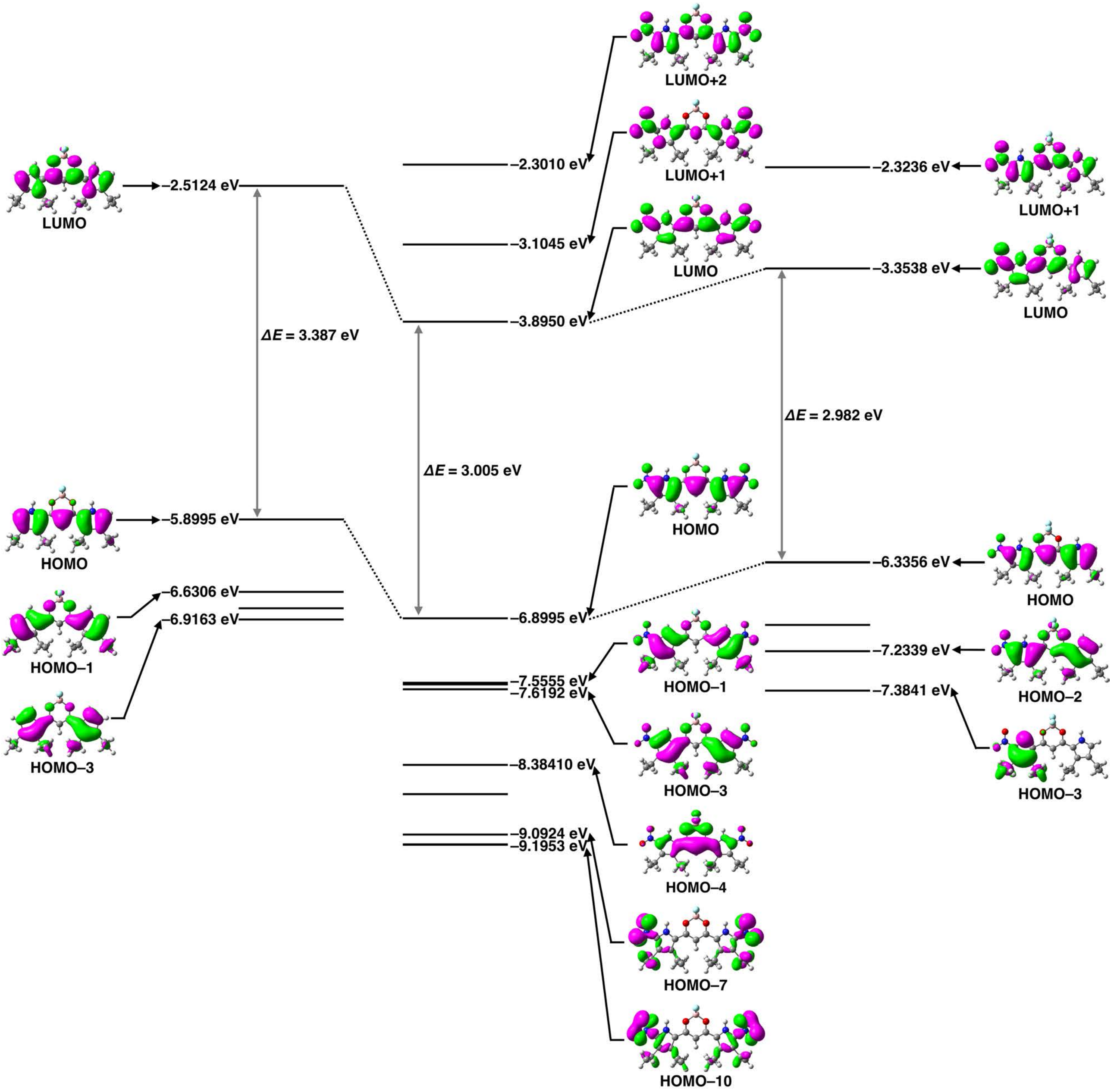

Figure S7 Molecular orbitals (HOMO/LUMO) of **1b** (left), **2a** (center), and **2b** (right) estimated at CPCM-B3LYP/6-31+G(d,p)(CH_2Cl_2)//B3LYP/6-31G(d,p).

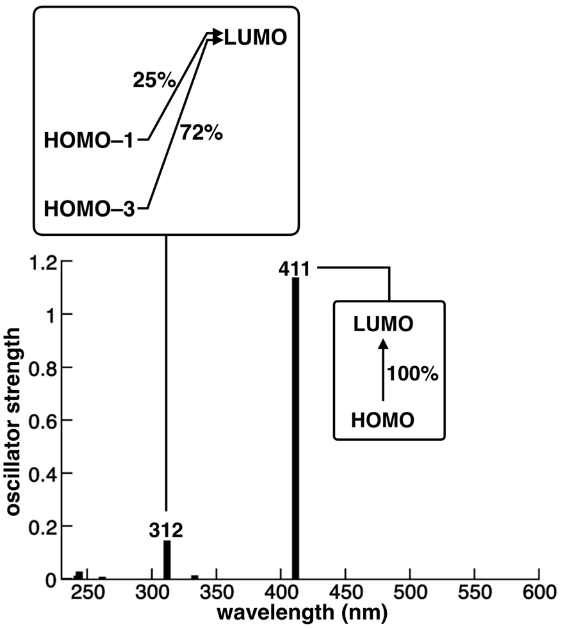

Figure S8 TD-DFT-based UV/vis absorption stick spectrum of **1b** with the transitions correlated with MOs estimated at CPCM-B3LYP/6-31+G(d,p)(CH_2Cl_2)//B3LYP/6-31G(d,p).

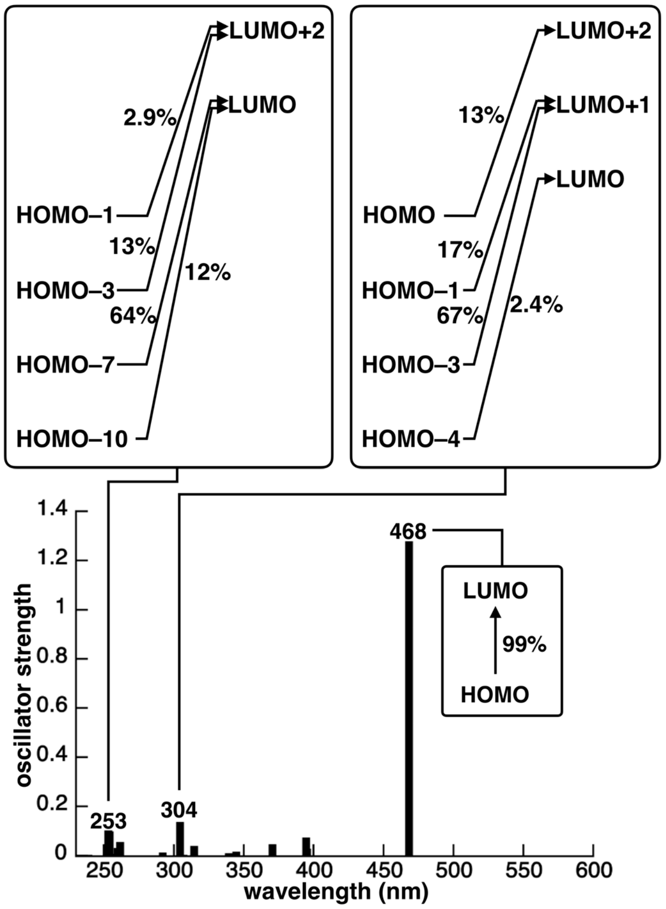

Figure S9 TD-DFT-based UV/vis absorption stick spectrum of **2a** with the transitions correlated with molecular orbitals (MOs) estimated at CPCM-B3LYP/6-31+G(d,p)(CH_2Cl_2)//B3LYP/6-31G(d,p).

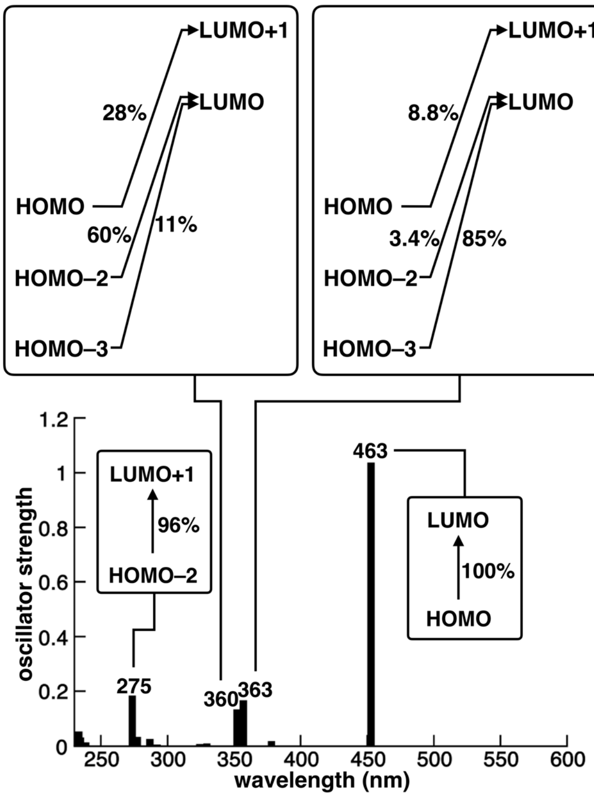

Figure S10 TD-DFT-based UV/vis absorption stick spectrum of **2b** with the transitions correlated with MOs estimated at CPCM-B3LYP/6-31+G(d,p)(CH_2Cl_2)//B3LYP/6-31G(d,p).

Cartesian Coordination of 2a-1
B3LYP/6-31G(d,p)
-1632.8225545 hartree
C,-0.0000166222,0.3064984509,-0.0001458363
C,1.2042705601,1.0184444727,-0.0143023756
C,-1.2043298961,1.0183968095,0.0142252719
H,0.0000058911,-0.7674205093,-0.0002433649
O,-1.2315707448,2.3204845377,0.0137310727
O,1.2314647906,2.320533109,-0.0135476115
C,-2.5258039607,0.4129754467,0.029571181
C,-3.024398838,-0.9110788428,0.0195762345
N,-3.5966600772,1.2643857594,0.0480084557
C,-4.4417367335,-0.8303439145,0.0342079109
C,-4.7382037617,0.537881172,0.0541902909
H,-3.5470103644,2.2752753197,0.0458233385
C,2.5257651553,0.4130690579,-0.0296335218
C,3.0243948671,-0.9109734502,-0.0198944246
N,3.5965977122,1.2645129013,-0.0478778857
C,4.4417333438,-0.8301952029,-0.0341850836
C,4.7381643562,0.5380424661,-0.0538871338
H,3.5469179021,2.2754007342,-0.0455877011
B,-0.0000723448,3.210584092,-0.0000723986
F,0.0127098455,3.9409148259,1.1490359291
F,-0.0128868451,3.940493843,-1.1494556109
O,-5.9381820156,2.4663649798,0.0282666168
O,5.9380787328,2.4665576011,-0.0273593533
C,-5.4277378127,-1.965623313,0.0411967766
H,-6.2597597163,-1.7147084707,-0.621692913
H,-4.946916763,-2.8586490935,-0.3707150167
C,-2.2322139481,-2.1911744195,0.0215253518
H,-2.8544828781,-2.9894428019,-0.3940737445
H,-1.3796554551,-2.105499252,-0.6605101641
C,2.2322426938,-2.1910885348,-0.022291752
H,2.8544408666,-2.9894242931,0.393282962
H,1.3795269611,-2.1055722817,0.659570066
C,5.4277685793,-1.9654452802,-0.0411607598
H,6.2596423237,-1.7146243141,0.6219501436
H,4.9468845738,-2.8585572426,0.3704908931
N,5.9863536829,1.230640609,-0.0561751949
N,-5.9864136192,1.2304407981,0.0568467914
O,-7.0222260624,0.5593435319,0.0874566652
O,7.0221929739,0.5595817959,-0.0867194633
C,-5.987382512,-2.2804097911,1.4406048888
H,-5.1891370019,-2.5439580157,2.1417205531
H,-6.5261724508,-1.4177602586,1.8397506338
H,-6.6856699464,-3.1211767314,1.3890583896
C,-1.7492464652,-2.616219921,1.4217610118
H,-1.1108521683,-1.8524147803,1.876582631
H,-2.5973837997,-2.7753769068,2.093787489
H,-1.18157187,-3.5505573393,1.3682748557
C,1.7496112834,-2.6158687764,-1.4227255729
H,1.1113049681,-1.8519920734,-1.8775492174
H,2.5979100358,-2.7748703534,-2.0945849193
H,1.1819487981,-3.5502320971,-1.3695591518
C,5.9877187652,-2.2799706287,-1.440505035
H,5.1896296477,-2.5434236177,-2.1418346521
H,6.5265649641,-1.4172349627,-1.8393887677
H,6.686022399,-3.1207238433,-1.3889557783

Cartesian Coordination of 2a-2
B3LYP/6-31G(d,p)
-1632.821874 hartree

C,-0.0188067081,0.1531405798,-0.4418761446
C,1.1724842275,0.8585948701,-0.2157521107
C,-1.2374132357,0.8032738484,-0.2304467683
H,0.0010541996,-0.8832338754,-0.7333032688
O,-1.2846765691,2.0683369575,0.0843734135
O,1.1729530056,2.1217824647,0.080776213
C,-2.5402896092,0.1662022488,-0.3011203304
C,-2.9993853544,-1.1435466601,-0.5758574711
N,-3.6327735656,0.9531137501,-0.0594862207
C,-4.4160971484,-1.1223626665,-0.4886255061
C,-4.7511963135,0.1980559107,-0.1635628743
H,-3.6130561584,1.942294477,0.1536841179
C,2.47318631,0.220346477,-0.2692469356
C,3.7655623272,0.7834259042,-0.3424624088
N,2.6012143153,-1.1458540026,-0.2985623118
C,4.6946499563,-0.2906877772,-0.4097391486
C,3.9223736585,-1.4552069902,-0.368008666
H,1.8917336105,-1.8561763535,-0.1906491403
B,-0.0761732478,2.9840586481,-0.0246188755
F,-0.0919334258,3.839317586,1.0289624717
F,-0.0863894905,3.5720577258,-1.2574110659
O,-6.0058611516,2.0401675129,0.2740088559
O,3.3482731435,-3.6516441232,-0.3537015704
C,-5.366153997,-2.2715386991,-0.6837651181
H,-6.2380021394,-1.9193368328,-1.240784229
H,-4.8796935381,-3.0361922873,-1.2977981705
C,-2.1690257804,-2.360702446,-0.8875802473
H,-2.7937797125,-3.0852071206,-1.4179802247
H,-1.3731782167,-2.0991370058,-1.5940222013
C,4.1069205629,2.2492399643,-0.362984168
H,5.0450529545,2.382989014,-0.9119041335
H,3.3359287104,2.7934546142,-0.9137160907
C,6.1926229777,-0.2027405112,-0.4963958655
H,6.5522504349,-0.9479451417,-1.2111059343
H,6.4658280211,0.7814697519,-0.8888759157
N,4.2809778341,-2.8333312865,-0.3939796939
N,-6.0172137856,0.8255916012,0.0378948453
O,-7.0306461169,0.1244144707,-0.0346089431
O,5.4772375772,-3.1268115763,-0.4544759146
C,-5.8431733469,-2.8996739807,0.6386586274
H,-5.0017748364,-3.2718180497,1.2318350708
H,-6.3886600079,-2.1654136079,1.2363467072
H,-6.5152866134,-3.739412772,0.4382658758
C,-1.5710848689,-3.0420004095,0.3584081334
H,-0.9343711182,-2.3564830288,0.9268707641
H,-2.3625771622,-3.3824527442,1.0320487133
H,-0.9722756037,-3.9128790941,0.0738570974
C,4.2443120197,2.8665205592,1.0415224271
H,3.297517529,2.8049455851,1.5828554891
H,5.0144316368,2.3565573302,1.6289057165
H,4.5208339275,3.9221875391,0.9633509525
C,6.8946739678,-0.4310032721,0.8552768437
H,6.5689097762,0.2988742972,1.6026794493
H,6.6815024018,-1.4335497176,1.2341869541
H,7.9783817374,-0.3370736557,0.7379429292

Cartesian Coordination of 2a-3
B3LYP/6-31G(d,p)
-1632.8194503 hartree
C,0.0000001323,0.0163240077,0.0000050934
C,1.2032701868,0.7269161651,-0.103510099
C,-1.2032683445,0.7269184985,0.1035241295
H,-0.0000005133,-1.0637739162,0.0000029155
O,-1.2274699668,2.023814753,0.1277310019
O,1.2274745,2.0238121088,-0.127728379
C,-2.4971202607,0.077474365,0.1923053028
C,-3.7654920576,0.6139470559,0.5052213794
N,-2.6439040513,-1.2728872557,-0.0061806056
C,-4.6987586287,-0.4585255937,0.4835533291
C,-3.9544465091,-1.5939227625,0.1523872355
H,-1.9645838805,-1.9565831105,-0.3069545186
C,2.4971209966,0.0774693168,-0.1922887024
C,3.7654919363,0.6139372801,-0.5052151684
N,2.6439054451,-1.2728887758,0.0062204098
C,4.6987537193,-0.4585399419,-0.4835615114
C,3.9544428595,-1.5939318799,-0.1523748357
H,1.9645888459,-1.956577499,0.3070187463
B,0.000000966,2.9080478605,-0.0000203021
F,0.1238216927,3.6395958484,1.1434244586
F,-0.1238219209,3.6395402961,-1.1435010976
O,-3.4199119389,-3.7507732669,-0.3119985364
O,3.4199101221,-3.7507741246,0.3120500308
C,-6.1771224809,-0.3932033542,0.7468718519
H,-6.4712597257,-1.2626733972,1.3407259304
H,-6.3895921247,0.4965787298,1.3472781409
C,-4.0856995633,2.0523837784,0.8131945122
H,-4.9823603781,2.0831649346,1.4408790229
H,-3.2728525268,2.4886825407,1.3984415832
C,4.0857000559,2.0523718365,-0.8131980971
H,4.9823444691,2.0831462611,-1.4409062569
H,3.2728403163,2.488674765,-1.3984248727
C,6.177112855,-0.3932256979,-0.746908565
H,6.4712350129,-1.2626989325,-1.3407656051
H,6.3895751635,0.4965535904,-1.3473215661
N,4.330075739,-2.9564218627,0.0275465452
N,-4.3300818974,-2.9564123496,-0.027533181
O,-5.5176910939,-3.2593448337,0.1078271235
O,5.5176830163,-3.2593580021,-0.107822494
C,-7.0229445268,-0.3598971089,-0.5397129486
H,-6.7652429162,0.500932645,-1.1642829575
H,-6.8690367446,-1.2701858153,-1.1243706391
H,-8.0862076221,-0.2935137571,-0.2906676365
C,-4.3133744898,2.915050919,-0.4426801622
H,-3.4054155361,2.9661869059,-1.0481286422
H,-5.1220649494,2.5138001776,-1.0617631256
H,-4.5827168533,3.9352200556,-0.1530496264
C,4.3134145864,2.9150399114,0.442668907
H,3.4054729025,2.9661811648,1.0481425159
H,5.1221203167,2.5137859375,1.0617298517
H,4.5827542825,3.9352072704,0.1530294936
C,7.0229595669,-0.359919294,0.5396599814
H,6.7652738414,0.5009136255,1.164232174
H,6.8690587933,-1.2702054674,1.1243233961
H,8.0862181813,-0.2935416062,0.2905940692

Cartesian Coordination of 2a·Cl⁻
B3LYP/6-31+G(d,p)
-2093.2270095 hartree
C,0.0143654039,0.2864950347,0.2704521881
C,-1.2017795569,0.95528789,0.1303693795

C,1.1932722633,0.962928448,-0.0408850268
H,0.0311634201,-0.7798308757,0.459980764
O,1.1940521004,2.2518106975,-0.2891389489
O,-1.246437416,2.2437926866,-0.1165941481
C,2.4762655201,0.2922284841,-0.1504030939
C,3.7709642851,0.859133063,-0.2518734444
N,2.5897183427,-1.0775827132,-0.1471513532
C,4.6917295304,-0.2124978513,-0.3047554912
C,3.9111968454,-1.3809122868,-0.2398462369
H,1.8069530013,-1.778427596,-0.1581767466
C,-2.4821875819,0.2760490019,0.2015050827
C,-3.7823493295,0.8354537606,0.2644071986
N,-2.5847783407,-1.0939414998,0.2456324703
C,-4.6958810709,-0.2413309921,0.339580408
C,-3.9055793309,-1.4042524255,0.3189575717
H,-1.8055480487,-1.7913960806,0.1373546079
B,-0.0120415153,3.0844554016,0.0257977416
F,-0.0805034984,4.1248028529,-0.8765067274
F,0.0810978849,3.5243835031,1.3446561
O,3.4687739023,-3.6214723352,-0.2821072529
O,-3.4505159684,-3.6352703235,0.5069046074
C,6.1899236932,-0.1112950896,-0.4000443014
H,6.5613948868,-0.8861601863,-1.0756145269
H,6.4477115871,0.8569440636,-0.8425849777
C,4.12507679,2.3244690367,-0.2616570083
H,5.1259888682,2.4378033887,-0.6923335729
H,3.434869078,2.8639913895,-0.91382571
C,-4.1444832053,2.2981674223,0.2769162341
H,-5.0897957801,2.4200740084,0.8183789954
H,-3.3861023025,2.8567328785,0.8305363224
C,-6.1943634205,-0.1468536938,0.4343970728
H,-6.5611551483,-0.9053135323,1.1314368534
H,-6.4571900104,0.8320656823,0.8501912529
N,-4.3200033391,-2.7673135352,0.4042250729
N,4.3339552951,-2.7431080854,-0.2822164278
O,5.5601992863,-2.9623040379,-0.3156409265
O,-5.5443838824,-2.9963546004,0.3737879619
C,6.9061384035,-0.2535310885,0.9568904509
H,6.5733886274,0.5145763751,1.6632724592
H,6.7070385284,-1.2354029215,1.3948919192
H,7.9901487553,-0.1525237703,0.8287837748
C,4.104052412,2.9789137534,1.13415422
H,3.1005316705,2.9474984729,1.5667842954
H,4.791700113,2.4721134004,1.8202466141
H,4.4045892783,4.0306122622,1.0648504598
C,-4.285846852,2.9176381118,-1.1272053188
H,-3.33815409,2.861236989,-1.6685373548
H,-5.0529034252,2.401407213,-1.7148138262
H,-4.5682762321,3.9738246913,-1.0501981594
C,-6.9128569586,-0.3312074138,-0.9164904115
H,-6.5868903398,0.4188554889,-1.6448461955
H,-6.7080375129,-1.3236173993,-1.3264900453
H,-7.9970697436,-0.2328755757,-0.7878260747
Cl,-0.0152298728,-2.9417205422,-0.1819487706

Cartesian Coordination of 2b-1

B3LYP/6-31G(d,p)

-1428.3269845 hartree

C,-0.0140630421,0.0635056091,-0.1056061437
C,-1.2271741631,0.7781227177,-0.0695253937
C,1.1811714007,0.7589088646,0.0488979512
H,-0.0175573553,-1.0065652684,-0.204218654
O,1.2134549441,2.0631519358,0.1567005208
O,-1.2321125414,2.0833853923,0.0181918597
C,2.4997620074,0.1455076141,0.1331152309
C,2.9929619511,-1.1793819776,0.1287052677
N,3.5667901347,0.9896235477,0.2614277609
C,4.4050774231,-1.1070838164,0.2578578113
C,4.7053694006,0.2584162902,0.3346847058
H,3.5137662418,1.9991289927,0.3055995758
C,-2.5368067905,0.1876810872,-0.1090095547
C,-3.0531390487,-1.1267642107,-0.1269247561
N,-3.6164499115,1.05073997,-0.1602279986
C,-4.4667934463,-1.0139906446,-0.1987807594
C,-4.7673562492,0.3476262984,-0.2190870386
H,-3.5010294031,2.0538406634,-0.1698306945
B,0.0132621914,2.9273783426,-0.1629179704
F,0.0881852498,3.3131588351,-1.4750580402
F,-0.0317328107,3.9530560996,0.7303166034
O,5.8989461061,2.175218962,0.5748068291
C,5.3833854273,-2.2483796818,0.2927188883
H,6.1412782387,-2.0419238743,1.0529391813
H,4.8585062159,-3.1588767891,0.5998020244
C,2.2005355747,-2.4516017667,-0.01029286
H,2.7751402849,-3.2730708457,0.4285035766
H,1.2842008655,-2.3872034012,0.5860063539
C,-2.2906197638,-2.4220541497,-0.045974267
H,-2.9413694168,-3.2317726387,-0.3919633195
H,-1.445282573,-2.4102409721,-0.7439479422
C,-5.4874716986,-2.1211828801,-0.2350765532
H,-6.3879912923,-1.7521105833,-0.739923825
H,-5.1174439568,-2.9469990285,-0.8545719062
N,5.9453918605,0.9405259439,0.4912629859
O,6.980187257,0.2659012385,0.5341967648
C,6.0881299884,-2.4911119831,-1.0545583799
H,5.367751414,-2.7071713409,-1.8499755773
H,6.6697179259,-1.6128892278,-1.3447831367
H,6.7735685075,-3.3406322865,-0.9767366706
C,1.8560424547,-2.8103909791,-1.4686278322
H,1.2658527566,-2.0231200662,-1.9477243516
H,2.765791174,-2.9438199226,-2.0612286616
H,1.2840383003,-3.7427660398,-1.5136929518
C,-1.7897737059,-2.7585903834,1.3719647559
H,-1.1177086808,-1.9840478644,1.7532949082
H,-2.6264556475,-2.8376775084,2.0719644744
H,-1.2524546748,-3.7125117125,1.3777505037
C,-5.8782354717,-2.6632800237,1.1514041024
H,-5.014748484,-3.0893738826,1.6714188265
H,-6.283913942,-1.8667024598,1.7829137411
H,-6.6375403103,-3.447115904,1.0630882095
H,-5.7272188168,0.8406695987,-0.2818696546

Cartesian Coordination of 2b-2

B3LYP/6-31G(d,p)

-1428.326323 hartree

C,-0.62437382,0.23830188,0.16148368
C,-1.82030621,0.97161455,0.26549119
C,0.59071428,0.89826833,0.34248005
H,-0.65258623,-0.81845686,-0.04600251
O,0.65798195,2.18681505,0.52544124

O,-1.7869137,2.2722631,0.42052806
C,1.86442459,0.19869974,0.34602622
C,3.17642093,0.6903565,0.17972419
N,1.93186761,-1.16437382,0.47128852
C,4.05526186,-0.42697542,0.21146793
C,3.2359592,-1.5445859,0.3999249
H,1.19555833,-1.81696326,0.69783945
C,-3.14128257,0.41058996,0.23772093
C,-3.67768027,-0.89517894,0.19586352
N,-4.20736591,1.28913376,0.30142159
C,-5.09080045,-0.76211695,0.23839642
C,-5.3702735,0.60271343,0.3044254
H,-4.07695421,2.28880034,0.35776079
B,-0.52046458,3.07266601,0.19209166
F,-0.52620735,4.13548485,1.03961481
F,-0.44000998,3.39918496,-1.14100994
O,2.57856372,-3.68185637,0.78776578
C,5.54946657,-0.42637697,0.04985496
H,5.98299672,-1.14261813,0.75275685
H,5.93429178,0.56267012,0.31610804
C,3.57738312,2.12581009,-0.04251755
H,4.65577735,2.21285147,0.12282801
H,3.09004744,2.75722944,0.70438142
C,-2.92732736,-2.1977711,0.12079351
H,-3.57236504,-2.9948911,0.50498131
H,-2.06379285,-2.17020866,0.79588559
C,-6.12857989,-1.85355389,0.20946717
H,-7.01632214,-1.51055727,0.75396875
H,-5.76150435,-2.72803587,0.75987075
N,3.53623911,-2.92447919,0.55691787
O,4.71222233,-3.28672776,0.45494957
C,6.00330247,-0.79050813,-1.37561552
H,5.58870533,-0.09725728,-2.11404101
H,5.68167912,-1.80296366,-1.63287232
H,7.09452978,-0.75326709,-1.44805539
C,3.2366068,2.66066732,-1.44723898
H,2.15608805,2.71136041,-1.60046325
H,3.67310129,2.03142069,-2.22985938
H,3.63467634,3.67310326,-1.56722875
C,-2.465346,-2.57926272,-1.29955989
H,-1.80129205,-1.82190377,-1.72733553
H,-3.32035574,-2.67713585,-1.97429
H,-1.93153634,-3.5348831,-1.2886463
C,-6.5474792,-2.2859838,-1.20712806
H,-5.69801821,-2.68478824,-1.76987447
H,-6.94951119,-1.43916051,-1.7721263
H,-7.31771309,-3.06289226,-1.16485476
H,-6.3237847,1.10902346,0.35813342

Cartesian Coordination of 2b-2′

B3LYP/6-31G(d,p)
-1428.3238603 hartree
C,0.0241432644,0.281895309,-0.0386577372
C,-1.1591016406,1.0450089315,0.0924830571
C,1.2520294431,0.9068999212,0.1431356068
H,-0.0213137687,-0.7662611231,-0.2846883286
O,1.3439065858,2.1999165405,0.3304262056
O,-1.1008667105,2.3283585673,0.3003889387
C,2.5410332143,0.2291538052,0.1493077118
C,2.9600110241,-1.1200355192,0.1494102362
N,3.6572858065,1.0157390747,0.18595277
C,4.3796628933,-1.1240897368,0.1777775637
C,4.7575805852,0.2237654905,0.1987710354
H,3.6617690612,2.0271999184,0.2165147195
C,-2.4697586401,0.4700446758,0.0256982823
C,-3.7294979778,1.0830192759,-0.1224076865
N,-2.6776816142,-0.9032840799,0.0590504651
C,-4.695889662,0.0410196605,-0.1627895125
C,-4.0075621645,-1.1599391452,-0.0313691266
H,-1.9737042081,-1.5876335592,0.2830321587
B,0.1671201146,3.1147928027,0.051830725
F,0.1895378827,3.4693841346,-1.2752756226
F,0.2134084829,4.1552042769,0.9237168599
O,6.0674149343,2.0752988155,0.3085835525
C,5.2935247837,-2.3179311182,0.1736957866
H,6.1054908951,-2.1463118758,0.8853213136
H,4.7365779075,-3.1931211882,0.524026593
C,2.0879113187,-2.3469641605,0.1350826472
H,2.6155540682,-3.1606407993,0.643162617
H,1.1891306564,-2.1626555735,0.7339828227
C,-4.0101268638,2.5555972831,-0.261616403
H,-5.0843422443,2.7161067809,-0.119668001
H,-3.4979267244,3.1014319389,0.5352750341
C,-6.1863248695,0.171247464,-0.3336823067
H,-6.6678620279,-0.7296698471,0.0648786316
H,-6.5545155698,1.0034232727,0.2780048038
N,6.0407486859,0.8386022849,0.2540333052
O,7.0374863226,0.1077707483,0.2459352726
C,5.9030071406,-2.6155672517,-1.2086192645
H,5.1262553309,-2.7961725776,-1.9585276758
H,6.5163619979,-1.7756881996,-1.5430162106
H,6.5412413321,-3.5031099068,-1.1592037076
C,1.6930036658,-2.8195354666,-1.2773590908
H,1.151838249,-2.0430973664,-1.827361865
H,2.5800715516,-3.073964391,-1.864265648
H,1.0575645094,-3.7098159934,-1.2257655291
C,-3.5843147535,3.1491471752,-1.619507313
H,-2.4994992871,3.1169073114,-1.7421722768
H,-4.0452532013,2.6080161728,-2.4523044493
H,-3.8944619225,4.1968587526,-1.6832296978
C,-6.6332334305,0.3782509509,-1.7916643345
H,-6.2095226883,1.2958514607,-2.2106031036
H,-6.3086822305,-0.4551329834,-2.4228002857
H,-7.7237118782,0.4500974539,-1.8572853882
H,-4.3865558196,-2.1714200679,0.0129484392

Cartesian Coordination of 2b-3

B3LYP/6-31G(d,p)
-1428.3223687 hartree
C,-0.0166825557,0.4971183153,0.0463376528
C,-1.2639464805,1.1492527828,0.1659191368
C,1.1487369608,1.2101794629,0.3190987946
H,0.0398764201,-0.5301805392,-0.2859537493
O,1.1324564644,2.4873129468,0.578151589
O,-1.3161019289,2.4179138296,0.4562918773
C,2.46011533,0.5810447828,0.3439995197
C,3.746583874,1.1421772309,0.2175711408
N,2.5961759913,-0.7778580994,0.4545787456
C,4.6839942804,0.0730935577,0.2633769707
C,3.9214799175,-1.0874572723,0.4231105021

H,1.8885469208,-1.4703354299,0.6493402108
C,-2.5201118068,0.481624929,-0.0027110541
C,-3.819172145,1.0048754353,-0.1576189036
N,-2.6178576868,-0.9018651617,-0.0874696295
C,-4.6954871707,-0.1018771701,-0.3256478953
C,-3.9174785554,-1.2524758695,-0.2601607186
H,-1.8698001845,-1.5484392078,0.1026541896
B,-0.1091043467,3.3176688863,0.3115114146
F,-0.0636815952,3.7633839315,-0.9876649874
F,-0.1809785172,4.2966824746,1.2509378792
O,3.3591586732,-3.2718616082,0.6712457979
C,6.1810799706,0.1644378431,0.1676890681
H,6.5474050129,-0.6450657785,-0.4696710253
H,6.445899273,1.1074890608,-0.3204135269
C,4.073077455,2.6012035813,0.0402935613
H,4.9900179141,2.6872248216,-0.553132239
H,3.2773230183,3.082228699,-0.5334396469
C,-4.2146368264,2.4571659538,-0.1861408765
H,-5.3015373203,2.5192687257,-0.0651604972
H,-3.7684862722,2.9732809281,0.6681413664
C,-6.183881381,-0.075909995,-0.5533237578
H,-6.6042992545,-1.0423959054,-0.2509984607
H,-6.6435210383,0.6704346829,0.1054913399
N,4.2870206213,-2.4546954079,0.5428708333
O,5.4855086043,-2.7490786479,0.5122932677
C,6.8853168376,0.0771006497,1.5345129956
H,6.5504828243,0.8727619536,2.207008067
H,6.6827658683,-0.8859106611,2.0095185835
H,7.9681681285,0.1713187717,1.4083810747
C,4.2514112517,3.3563344222,1.3702397411
H,3.3231414681,3.3406796276,1.946153036
H,5.0456956205,2.9131740976,1.9795206726
H,4.5138909471,4.4013700948,1.1792126673
C,-3.804733416,3.1937166665,-1.477188209
H,-2.7181331701,3.2556520108,-1.5689534954
H,-4.2007047573,2.6908016101,-2.3654226178
H,-4.1958331376,4.2159176732,-1.4645041149
C,-6.5885907573,0.2162315685,-2.0091092759
H,-6.2267940924,1.196709214,-2.3325559675
H,-6.170714779,-0.5326941421,-2.689269707
H,-7.6778133744,0.2057283907,-2.118997291
H,-4.2139776081,-2.2905554269,-0.3163891194

Cartesian Coordination of 2b·Cl⁻

B3LYP/6-31+G(d,p)

-1888.7218668 hartree

C,-0.4207560021,0.2907241599,0.2892811669
C,-1.6991084186,-0.249224414,0.0423847502
C,0.6862019404,-0.533958232,0.2180127883
H,-0.2962934355,1.3593698636,0.4098272915
O,0.5690090772,-1.8384528975,0.061398727
O,-1.8616764484,-1.546310641,-0.0882173418
C,2.0506413178,-0.0221781431,0.2679362631
C,3.2643581317,-0.7390983798,0.3867202274
N,2.3347573985,1.3208184785,0.2226585884
C,4.3135872372,0.2112862214,0.4077495622
C,3.688503183,1.4644087008,0.300807192
H,1.664583555,2.1088751781,0.1145525281
C,-2.8522191151,0.5816204119,-0.1269950352
C,-4.2190360656,0.2503944743,-0.2946654793
N,-2.7421882151,1.9638629841,-0.1684531521
C,-4.9185807767,1.4737054174,-0.4378461094
C,-3.9629840818,2.4960257877,-0.3595180672
H,-1.8616469095,2.5240356596,-0.1374545298
B,-0.7583392587,-2.497578219,0.2657257875
F,-0.8869134654,-2.8489860916,1.6109875007
F,-0.8399968601,-3.5961586508,-0.5700820096
O,3.5225521309,3.7395482538,0.2545377152
C,5.7853971178,-0.0764217347,0.5273110895
H,6.2457027894,0.6614867485,1.1900267481
H,5.9115251109,-1.0590215664,0.9950742637
C,3.4317322099,-2.2332698338,0.4951542659
H,4.3470414461,-2.4422303494,1.0615159982
H,2.6006806889,-2.65157087,1.0672712223
C,-4.8256704478,-1.127943075,-0.3015546184
H,-5.8219939607,-1.0681822919,-0.7570113141
H,-4.2243960815,-1.7901147234,-0.9316891452
C,-6.3975399266,1.6822535989,-0.6305605667
H,-6.555859512,2.6412094033,-1.1407155739
H,-6.7964934083,0.9134495259,-1.3050491863
N,4.2737486613,2.7597454342,0.2908667776
O,5.5189837769,2.8310342494,0.3205134322
C,6.5318670613,-0.0604669015,-0.8209043111
H,6.1100028172,-0.7943338308,-1.5157695556
H,6.4696559558,0.9286885258,-1.2825703395
H,7.5914103811,-0.3004541005,-0.6730576076
C,3.5056996654,-2.9551283251,-0.864210681
H,2.5751658419,-2.818297663,-1.4209377572
H,4.3343131275,-2.5761947404,-1.4728853952
H,3.6551293808,-4.0310536146,-0.7166151859
C,-4.9488676835,-1.7652703607,1.0971175046
H,-3.9624192195,-1.9062574728,1.5460965229
H,-5.546374114,-1.1376677088,1.7679253778
H,-5.4305056275,-2.7479128033,1.0285688992
C,-7.2140936845,1.6725983765,0.6763289934
H,-7.1147301124,0.714137247,1.1961777547
H,-6.8655593751,2.4554949797,1.3587021961
H,-8.279407767,1.8420550833,0.4761416252
H,-4.0882272523,3.5673231311,-0.4437119033
Cl,-0.0628599981,3.6206989702,-0.0870457448

Cartesian Coordination of 2c-1

B3LYP/6-31G(d,p) for C, H, N, and O and B3LYP/LanL2DZ for I

-1439.0747263 hartree

C,0.6373264605,0.2529801778,0.1288875968
C,1.7937802933,1.0539444664,0.0916527687
C,-0.6058794956,0.8601927399,-0.0335696631
H,0.7167307769,-0.8134787855,0.2342802075
O,-0.7316840434,2.1574850794,-0.1460886133
O,1.7060922149,2.3545630718,-0.0115503379
C,-1.8760480231,0.1526615336,-0.1148708817
C,-2.2707125483,-1.2049809188,-0.1353691837
N,-3.0045771424,0.9189788018,-0.2083350939
C,-3.6866737053,-1.2338765291,-0.2408621234
C,-4.0872032029,0.1073048648,-0.2805707295
H,-3.0271880147,1.9305008017,-0.230792725
C,3.1455910214,0.5626236418,0.1413531564
C,3.7630068292,-0.7034123746,0.2025457174
N,4.1538229393,1.5108554559,0.1500266525
C,5.1686907169,-0.4898454082,0.2544831018

C,5.3521476899,0.8936572229,0.2195992887
H,3.9662641074,2.5034304368,0.1270853749
B,0.4030789802,3.1081938064,0.1692332029
F,0.3018880156,3.4878270483,1.480973485
F,0.3740930562,4.1311553247,-0.7273610784
O,-5.4223489998,1.935456074,-0.4548682398
C,-4.5803016225,-2.4424650486,-0.2882770705
H,-5.3636599927,-2.2746614619,-1.0321473959
H,-3.9955878544,-3.305140017,-0.6242500949
C,-1.3847241527,-2.4190865378,-0.0477741435
H,-1.8922336865,-3.2604479238,-0.530323117
H,-0.4724177977,-2.2569082181,-0.631389283
C,3.0977198394,-2.0541127363,0.1857860812
H,3.7941262721,-2.7935196919,0.5939757418
H,2.2378775136,-2.0582936532,0.8651898013
C,6.2556798307,-1.5273188924,0.3222873548
H,7.1094334987,-1.1091189747,0.8670256919
H,5.9089102792,-2.38026659,0.9167651889
N,-5.3774575881,0.6994856761,-0.4011949717
O,-6.3589354557,-0.04979061,-0.4446872011
C,-5.2436380315,-2.7654597623,1.0636199965
H,-4.4966823866,-2.9458243936,1.8433948512
H,-5.8822796191,-1.9381331035,1.3824284554
H,-5.8671837715,-3.6606516143,0.9764253111
C,-1.0224665197,-2.8201025927,1.3955549793
H,-0.5022778731,-2.0121006671,1.919603494
H,-1.9222764013,-3.0562018091,1.9711756177
H,-0.3765267308,-3.7041249469,1.4015881707
C,2.6560245933,-2.5094447656,-1.2190581205
H,1.9436884261,-1.8071492425,-1.66259252
H,3.5132073501,-2.5755157848,-1.8952709775
H,2.1833317103,-3.4960223802,-1.1751078683
C,6.7332397753,-2.0253779829,-1.0541498343
H,5.9171462525,-2.4924237893,-1.614011368
H,7.1203328345,-1.1989301129,-1.657669595
H,7.53172316,-2.7657000003,-0.9409536563
I,7.1344747518,2.005287956,0.2790541587

Cartesian Coordination of 2c-2

B3LYP/6-31G(d,p) for C, H, N, and O and B3LYP/LanL2DZ for I
-1439.0741609 hartree
C,-0.6271798649,0.2182100202,-0.1270700139
C,-1.817485961,0.9597247955,-0.2249047332
C,0.5923592283,0.8749516114,-0.3061191795
H,-0.6623829271,-0.8398574413,0.0749829018
O,0.6604097301,2.1631636308,-0.4875307588
O,-1.7809735775,2.2613108687,-0.3639770721
C,1.8656864436,0.1766035361,-0.3098159862
C,3.1792499242,0.6795867421,-0.18888661
N,1.9376599993,-1.1905046772,-0.3872757208
C,4.0628678719,-0.4346507637,-0.197305183
C,3.2448442723,-1.5615556789,-0.3263795034
H,1.2010576452,-1.8578348301,-0.5645259606
C,-3.1446927926,0.4074717471,-0.2028245996
C,-3.6969059812,-0.8892990158,-0.2056890849
N,-4.1989853827,1.3034351623,-0.2212989674
C,-5.1127125399,-0.7490687236,-0.225401836
C,-5.3655958101,0.6240186856,-0.2350647333
H,-4.0620881794,2.3041242677,-0.2442281497
B,-0.5088577259,3.0535109232,-0.1363594191
F,-0.5152048219,4.1242904373,-0.9735939212
F,-0.4169668536,3.3628262946,1.1996354484
O,2.5895071527,-3.7150228915,-0.6137501386
C,5.5605796798,-0.420113078,-0.0712160356
H,5.9813910724,-1.1724738328,-0.7433999722
H,5.9341786909,0.5543246501,-0.4004842758
C,3.5799470021,2.1232872906,-0.0290843595
H,4.6498328806,2.2114373509,-0.2432218523
H,3.0562902442,2.7291696885,-0.7722914005
C,-2.9549150332,-2.1992316026,-0.1971235451
H,-3.5938567738,-2.969831811,-0.6406309162
H,-2.0805293598,-2.1302866087,-0.8547058436
C,-6.1471412552,-1.8408466097,-0.2311417382
H,-7.0177609723,-1.5005791277,-0.8030578419
H,-5.7547694813,-2.7108042527,-0.7696526345
N,3.5484289672,-2.9467646309,-0.4311129859
O,4.7272245414,-3.2990508606,-0.333703362
C,6.0497664663,-0.7029066741,1.3615716582
H,5.654260502,0.0321853503,2.0695947256
H,5.7340646095,-1.6984741349,1.6843733402
H,7.1425649786,-0.6636534129,1.4046100881
C,3.2954731296,2.696514781,1.3735847868
H,2.2219994189,2.7410222492,1.5726705735
H,3.7720918884,2.0934507112,2.1535471529
H,3.6881296016,3.7155514964,1.4471248414
C,-2.5178912076,-2.6651813115,1.2060231295
H,-1.8644779495,-1.9328054884,1.6905457518
H,-3.3845249886,-2.8066263143,1.8579975601
H,-1.9804596843,-3.6170420519,1.1465319133
C,-6.6072412791,-2.2759484791,1.1725386252
H,-5.7718007141,-2.6640570027,1.7631886324
H,-7.040603693,-1.4348649008,1.7219579226
H,-7.3656832934,-3.0622927369,1.1030284969
I,-7.2033952778,1.6420826632,-0.280246864

Cartesian Coordination of 2c-2´

B3LYP/6-31G(d,p) for C, H, N, and O and B3LYP/LanL2DZ for I
-1439.0721455 hartree
C,-0.4310646997,0.3022702403,-0.2124897841
C,-1.7075212327,-0.2806809454,-0.3710110669
C,0.6922088667,-0.51792696,-0.252713696
H,-0.3250894717,1.3630678034,-0.0543300554
O,0.587423593,-1.8206092006,-0.3272932864
O,-1.8437020402,-1.5705768108,-0.4739490243
C,2.0691350019,-0.0478469178,-0.220539767
C,2.694507612,1.2164006157,-0.3068705346
N,3.0483920243,-0.9946892037,-0.1072508829
C,4.0966194629,1.0025174537,-0.2328395046
C,4.2580126149,-0.3826886944,-0.1085672726
H,2.8971471002,-1.9936782885,-0.049027074
C,-2.9146445841,0.4915676878,-0.4451822317
C,-4.2602346464,0.0969325161,-0.3385198721
N,-2.8964866505,1.8731448712,-0.5992095183
C,-5.0614886779,1.2702998747,-0.4463493962
C,-4.1753511139,2.3284406,-0.6214325589
H,-2.0879191393,2.4261893151,-0.8349230723
B,-0.7345161358,-2.5145585782,-0.0622733193
F,-0.8512579222,-2.7354843583,1.2886825294
F,-0.8004807294,-3.6289579712,-0.8359437632
O,5.2631387075,-2.4128383702,0.0415583461

C,5.1855126892,2.0388702402,-0.2698857112
H,6.0019781615,1.674219298,-0.8988638291
H,4.7958070913,2.9473730607,-0.7410055104
C,2.0283922964,2.557821652,-0.4598891981
H,2.7082752162,3.2296636468,-0.9939027965
H,1.1491004229,2.4585302875,-1.1061558002
C,-4.7779400261,-1.2971314085,-0.097875276
H,-5.853291604,-1.3067517471,-0.3038144073
H,-4.3101447446,-1.9845487681,-0.8077219225
C,-6.5599690687,1.3631777972,-0.3654576617
H,-6.8913262185,2.2473592169,-0.9208021388
H,-7.002667496,0.5007489202,-0.8762877137
N,5.4299988968,-1.1875349563,-0.0144930574
O,6.5261882925,-0.6179157769,0.0036612835
C,5.7497680296,2.3825664905,1.1215656303
H,4.9669832634,2.7528379318,1.7914253986
H,6.2041692005,1.499497968,1.5771943201
H,6.5200592092,3.155549585,1.0385864332
C,1.6305944963,3.215248011,0.8760314615
H,0.9457359516,2.5830290496,1.4502809703
H,2.5113925279,3.3895053124,1.500724372
H,1.1437389514,4.1808971029,0.7041753196
C,-4.5312778707,-1.8172324292,1.3329507563
H,-3.4641584952,-1.9397023625,1.5312915281
H,-4.9497096055,-1.1365032,2.0815421418
H,-5.0069603532,-2.7946508913,1.4608964048
C,-7.1011711777,1.4369064413,1.0738815493
H,-6.8223910617,0.548722099,1.6490042976
H,-6.7045680278,2.3121368052,1.5976427068
H,-8.1934877672,1.5090427095,1.0715842025
I,-4.5740550191,4.3747215446,-0.902719778

Cartesian Coordination of 2c-3

B3LYP/6-31G(d,p) for C, H, N, and O and B3LYP/LanL2DZ for I
-1439.0705267 hartree
C,-0.4271179595,0.3716475959,-0.0023170172
C,-1.7133993413,-0.133915505,-0.2860489715
C,0.6681076933,-0.4897434548,-0.073549324
H,-0.2918548727,1.4017896492,0.2973824598
O,0.5260565643,-1.7668483405,-0.2878872788
O,-1.8843496131,-1.3996492026,-0.540282239
C,2.0382684383,-0.0271391103,0.0743334201
C,3.2135621779,-0.7397569693,0.3889914146
N,2.3621392295,1.2998332544,-0.0424553127
C,4.27829321,0.2017494817,0.4534002523
C,3.6994075439,1.4417291288,0.1679537227
H,1.7829951036,2.0703887878,-0.3415134905
C,-2.8906866603,0.6848474625,-0.3297945449
C,-4.2508791086,0.3275233679,-0.3526920143
N,-2.825779529,2.0736008135,-0.3077796016
C,-5.0114219151,1.5322423869,-0.3581114251
C,-4.0877890418,2.572675768,-0.3454999335
H,-1.9904340746,2.6258274446,-0.4178723168
B,-0.8350524887,-2.4270924405,-0.1772302544
F,-1.0354617831,-2.7932236665,1.1320264936
F,-0.8935085034,-3.4452739762,-1.0750989908
O,3.4619263359,3.6688830842,-0.194637364
C,5.7249635319,-0.0714931516,0.7560061681
H,6.102010696,0.7059379411,1.4264058864
H,5.7998431592,-1.0246564177,1.2886650139
C,3.3244989144,-2.2203230609,0.6393599201
H,4.1424371798,-2.3969213582,1.3465537506
H,2.4084859586,-2.5754152036,1.1178876747
C,-4.8209679674,-1.0665483064,-0.324837678
H,-5.8780634605,-1.0127829503,-0.6050612042
H,-4.3204498833,-1.6771050367,-1.0809554389
C,-6.5088811249,1.6672706387,-0.3560318468
H,-6.7792517833,2.6234931159,-0.8170657942
H,-6.942026956,0.8896461091,-0.9950855327
N,4.251337006,2.7484148783,0.0758326998
O,5.4622478759,2.8896784211,0.2675327719
C,6.6118457599,-0.1143161603,-0.5028334694
H,6.2713618442,-0.8824029384,-1.2041588405
H,6.5987003992,0.851756138,-1.0134880955
H,7.6472660793,-0.3385274845,-0.2286010533
C,3.5676050525,-3.043521363,-0.6396447688
H,2.7294968574,-2.9317025289,-1.3314713178
H,4.4839920929,-2.7298399872,-1.1501778034
H,3.6637904092,-4.1053931293,-0.3928751642
C,-4.6933784406,-1.7675702217,1.0429577646
H,-3.6468096881,-1.9449070307,1.3008089572
H,-5.1531083349,-1.1736286481,1.8396300155
H,-5.1970522827,-2.738846424,1.0115584611
C,-7.1388105476,1.5847128263,1.0462440059
H,-6.9262081287,0.6220969987,1.5211050986
H,-6.7489600029,2.3721407389,1.698607944
H,-8.225864116,1.6998282864,0.987627861
I,-4.4127726145,4.6502297692,-0.3876218595

4. Anion-binding behaviors

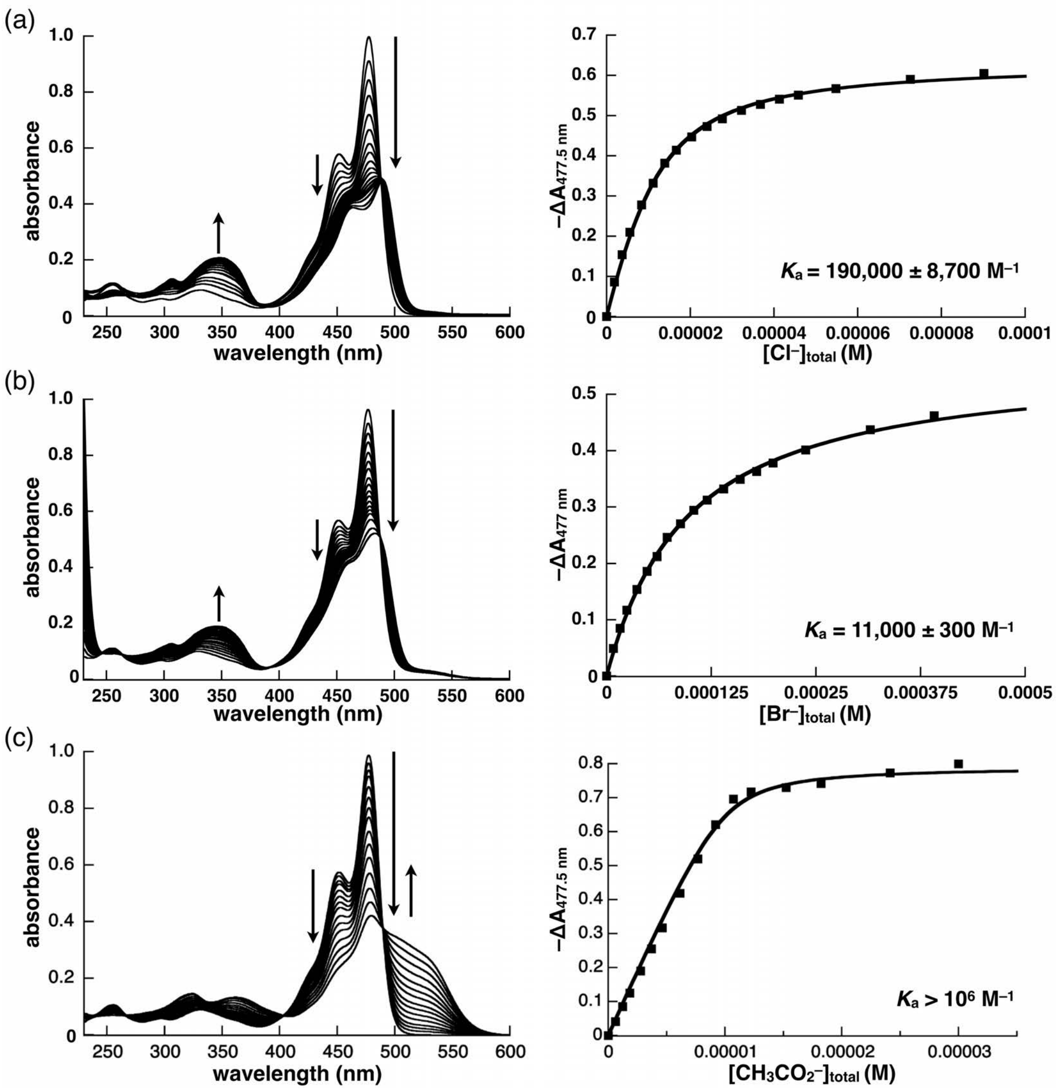

Figure S11 UV/vis absorption spectral changes (left) and titration plots and 1:1 fitting curves (right) of **2a** (1.0×10^{-5} M) upon the addition of (a) Cl^-, (b) Br^-, and (c) $CH_3CO_2^-$ as tetrabutylammonium (TBA) salts in CH_2Cl_2. Under this condition, the K_a value for $CH_3CO_2^-$ is too large to be exactly estimated. The spectral changes along with the red shift upon the addition of $CH_3CO_2^-$ were probably derived from the interactions between pyrrole NH and $CH_3CO_2^-$ with strong basicity.

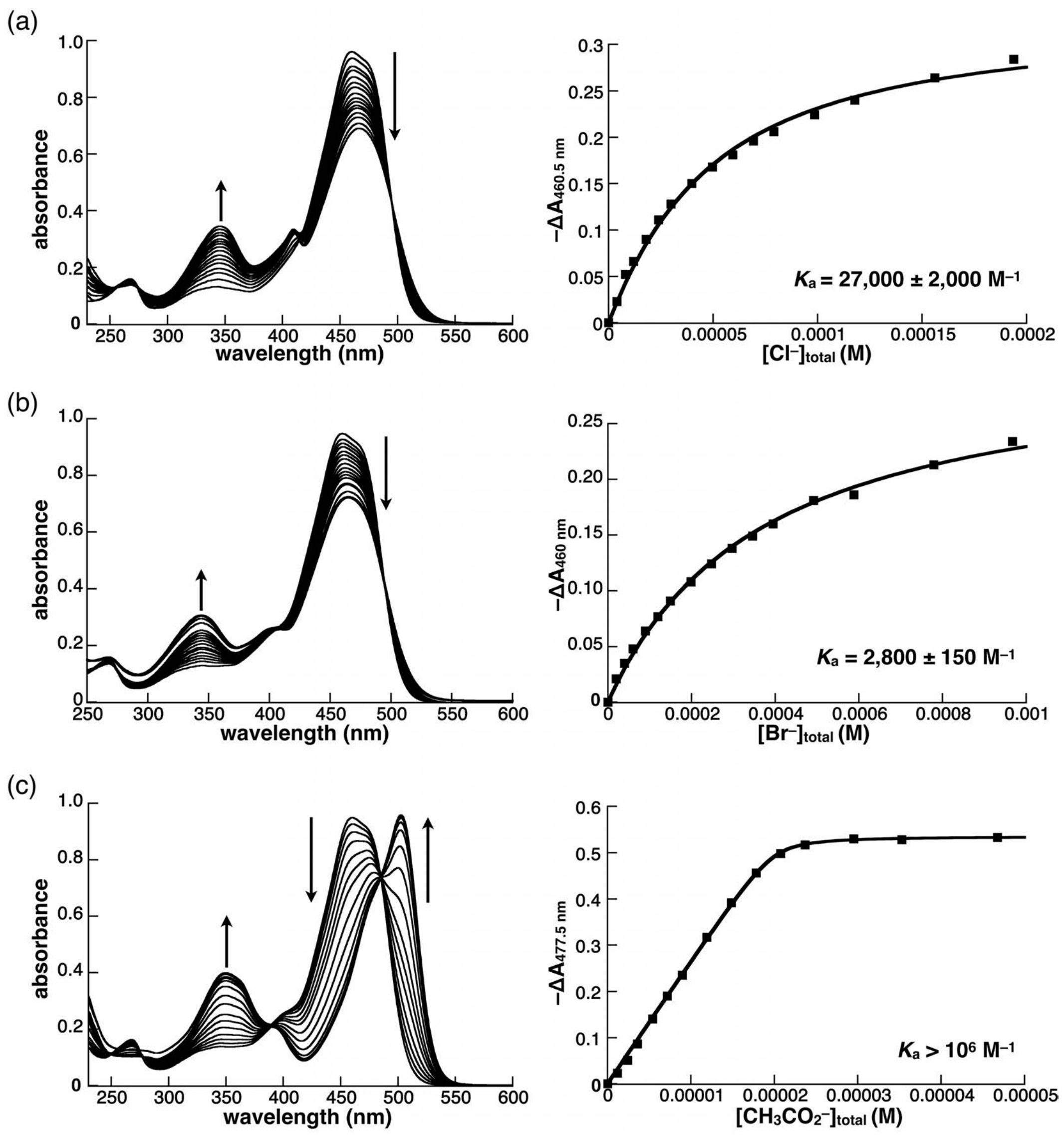

Figure S12 UV/vis absorption spectral changes (left) and titration plots and 1:1 fitting curves (right) of **2b** (2.0×10^{-5} M) upon the addition of (a) Cl^- , (b) Br^-, and (c) $CH_3CO_2^-$ as TBA salts in CH_2Cl_2. Under this condition, the K_a value for $CH_3CO_2^-$ is too large to be exactly estimated. The spectral changes along with the red shift upon the addition of $CH_3CO_2^-$ were probably derived from the interactions between pyrrole NH and $CH_3CO_2^-$ with strong basicity.

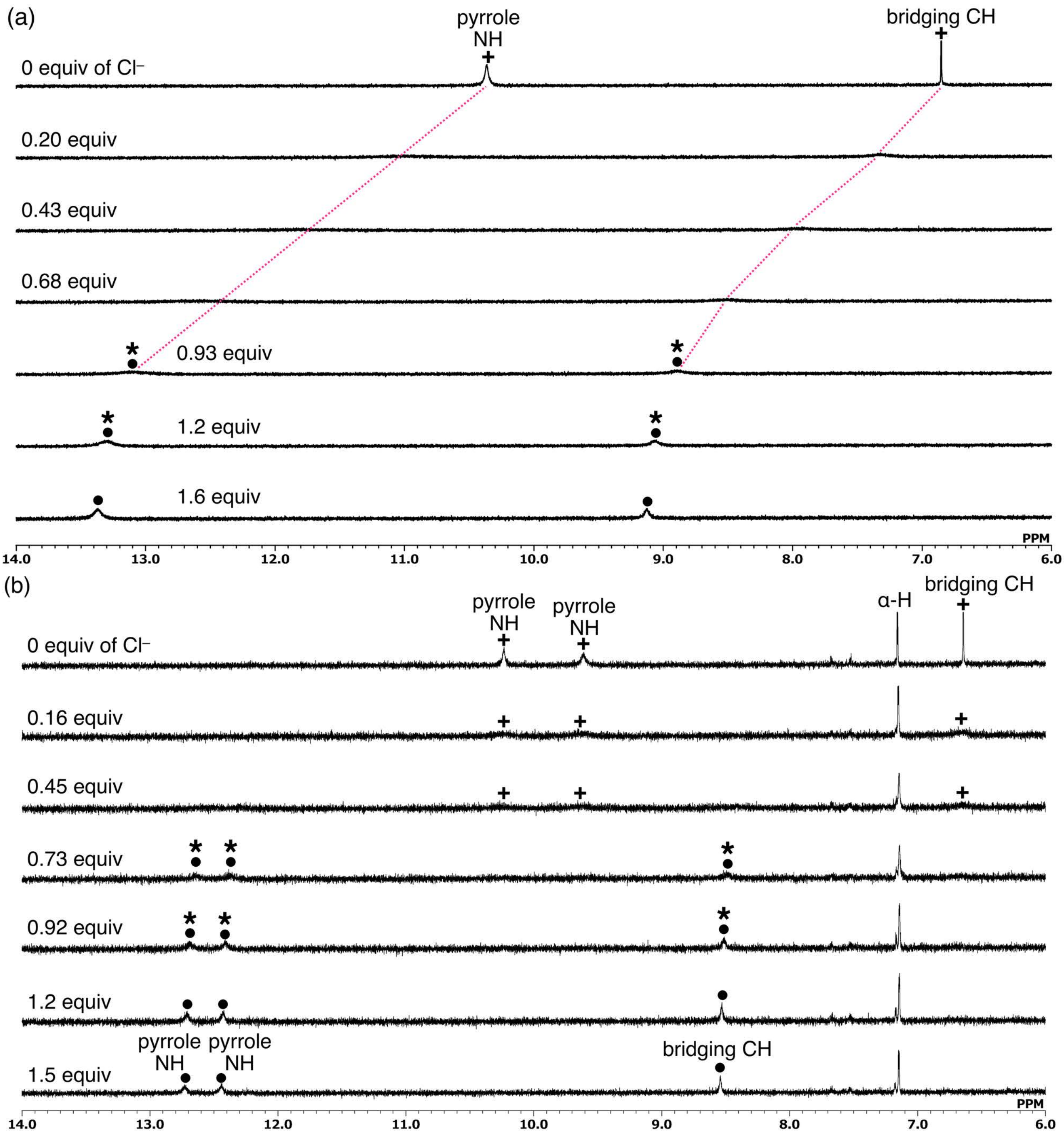

Figure S13 ^{1}H NMR spectral changes of (a) **2a** (1×10^{-3} M) upon the addition of Cl^- (0–1.6 equiv) added as a TBA salt and (b) **2b** (1×10^{-3} M) upon the addition of Cl^- (0–1.5 equiv) added as a TBA salt in CD_2Cl_2 at 20 °C. The signals of anion-free receptors, [1+1]-type, and [2+1]-type complexes are labeled by plus, circle, and asterisk marks, respectively.
